# GE23077 binds to the RNA polymerase ‘i’ and ‘i+1’ sites and prevents the binding of initiating nucleotides

**DOI:** 10.7554/eLife.02450

**Published:** 2014-04-22

**Authors:** Yu Zhang, David Degen, Mary X Ho, Elena Sineva, Katherine Y Ebright, Yon W Ebright, Vladimir Mekler, Hanif Vahedian-Movahed, Yu Feng, Ruiheng Yin, Steve Tuske, Herbert Irschik, Rolf Jansen, Sonia Maffioli, Stefano Donadio, Eddy Arnold, Richard H Ebright

**Affiliations:** 1 Waksman Institute, Rutgers University, Piscataway, United States; 2 Department of Chemistry and Chemical Biology, Rutgers University, Piscataway, United States; 3 Center for Advanced Biotechnology and Medicine, Rutgers University, Piscataway, United States; 4 Natural Products Chemistry, Helmholtz Centre for Infection Research, Braunschweig, Germany; 5 Microbial Drugs, Helmholtz Centre for Infection Research, Braunschweig, Germany; 6 Naicons Srl, Milan, Italy; National Institute of Child Health and Human Development, United States

**Keywords:** RNA polymerase, RNA polymerase-promoter open complex, transcription, transcription initiation, inhibitor, bipartite inhibitor, *E. coli*, *Streptococcus pyogenes*

## Abstract

Using a combination of genetic, biochemical, and structural approaches, we show that the cyclic-peptide antibiotic GE23077 (GE) binds directly to the bacterial RNA polymerase (RNAP) active-center ‘i’ and ‘i+1’ nucleotide binding sites, preventing the binding of initiating nucleotides, and thereby preventing transcription initiation. The target-based resistance spectrum for GE is unusually small, reflecting the fact that the GE binding site on RNAP includes residues of the RNAP active center that cannot be substituted without loss of RNAP activity. The GE binding site on RNAP is different from the rifamycin binding site. Accordingly, GE and rifamycins do not exhibit cross-resistance, and GE and a rifamycin can bind simultaneously to RNAP. The GE binding site on RNAP is immediately adjacent to the rifamycin binding site. Accordingly, covalent linkage of GE to a rifamycin provides a bipartite inhibitor having very high potency and very low susceptibility to target-based resistance.

**DOI:** http://dx.doi.org/10.7554/eLife.02450.001

## eLife digest

As increasing numbers of bacteria become resistant to antibiotics, new drugs are needed to fight bacterial infections. To develop new antibacterial drugs, researchers need to understand how existing antibiotics work. There are many ways to kill bacteria, but one of the most effective is to target an enzyme called bacterial RNA polymerase. If bacterial RNA polymerase is prevented from working, bacteria cannot synthesize RNA and cannot survive.

GE23077 (GE for short) is an antibiotic produced by bacteria found in soil. Although GE stops bacterial RNA polymerase from working, and thereby kills bacteria, it does not affect mammalian RNA polymerases, and so does not kill mammalian cells. Understanding how GE works could help with the development of new antibacterial drugs.

Zhang et al. present results gathered from a range of techniques to show how GE inhibits bacterial RNA polymerase. These show that GE works by binding to a site on RNA polymerase that is different from the binding sites of previously characterized antibacterial drugs. The mechanism used to inhibit the RNA polymerase is also different.

The newly identified binding site has several features that make it an unusually attractive target for development of antibacterial compounds. Bacteria can become resistant to an antibiotic if genetic mutations lead to changes in the site the antibiotic binds to. However, the site that GE binds to on RNA polymerase is essential for RNA polymerase to function and so cannot readily be changed without crippling the enzyme. Therefore, this type of antibiotic resistance is less likely to develop.

In addition, the newly identified binding site for GE on RNA polymerase is located next to the binding site for a current antibacterial drug, rifampin. Zhang et al. therefore linked GE and rifampin to form a two-part (‘bipartite’) compound designed to bind simultaneously to the GE and the rifampin binding sites. This compound was able to inhibit drug-resistant RNA polymerases tens to thousands of times more potently than GE or rifampin alone.

**DOI:** http://dx.doi.org/10.7554/eLife.02450.002

## Introduction

**Figure 1.**
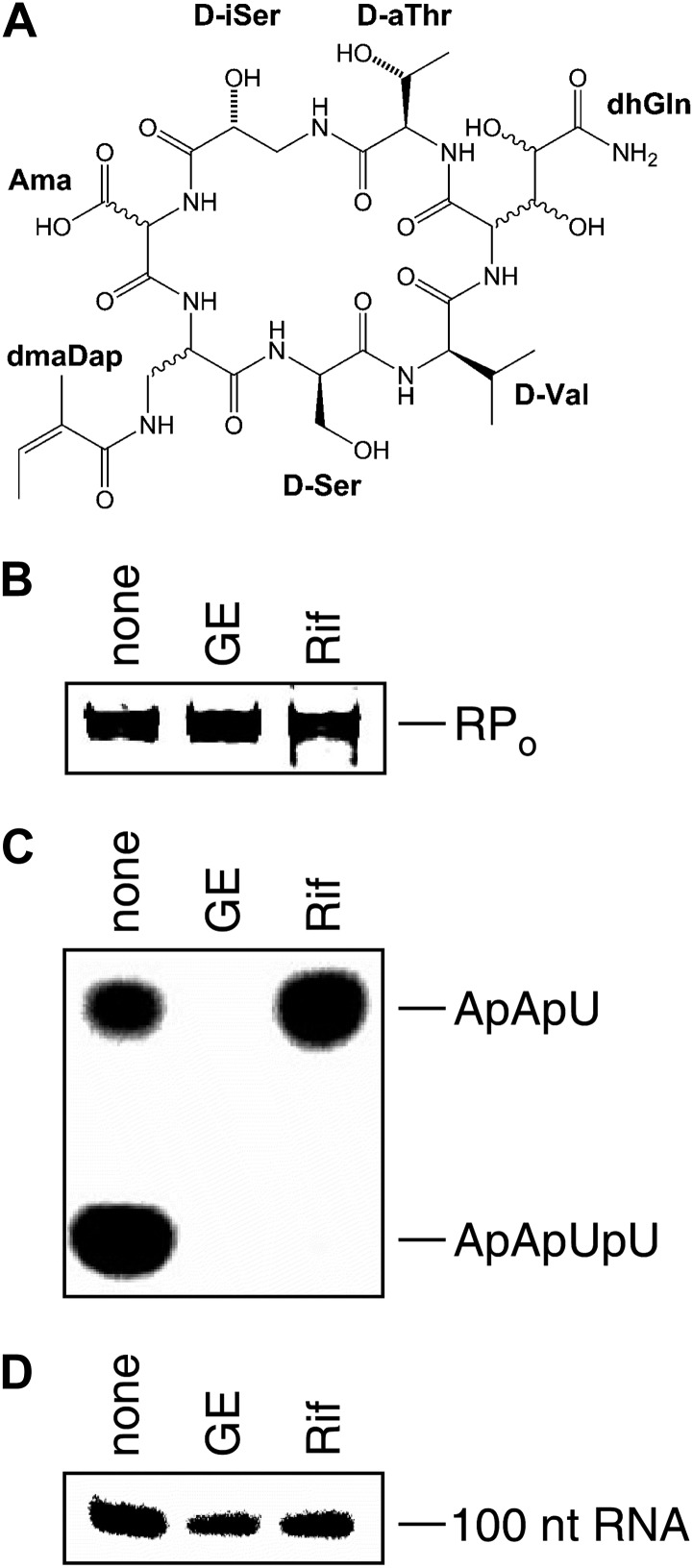
Mechanism of transcription inhibition by GE: inhibition of first nucleotide addition in transcription initiation. (**A**) Structure of GE. dmaDap, N^β^-(Z-2,3-dimethylacryloyl)-α,β-diaminopropionic acid; dhGln, β,γ-dihydroxy-glutamine; Ama, aminomalonic acid; aThr, allothreonine; iSer, isoserine. Wavy bonds, previously undefined stereochemistry. (**B**) GE does not inhibit formation of a transcription initiation complex. (**C**) GE inhibits nucleotide addition in transcription initiation (primer-dependent transcription initiation). (**D**) GE does not inhibit nucleotide addition in transcription elongation (elongation from halted TEC containing 29 nt RNA product). See Figure 1—figure supplements 1, 2. **DOI:** http://dx.doi.org/10.7554/eLife.02450.003

GE23077 (GE) is a cyclic-peptide antibiotic produced by the soil bacterium *Actinomadura* sp. DSMZ 13491 (Figure 1A; Ciciliato et al., 2004). GE exhibits antibacterial activity against both Gram-negative and Gram-positive bacterial pathogens in culture, including *Moraxella catarrhalis* and *Streptococcus pyogenes* (Supplementary file 1A; Ciciliato et al., 2004). GE inhibits both Gram-negative and Gram-positive bacterial RNA polymerase (RNAP) in vitro, but does not inhibit human RNAP I, II, or III in vitro (Supplementary file 1B; Ciciliato et al., 2004). Analysis of the kinetics of inhibition suggests that GE inhibits RNAP at a stage subsequent to the formation of the RNAP-template complex (Sarubbi et al., 2004).

**Figure 1—figure supplement 1.**
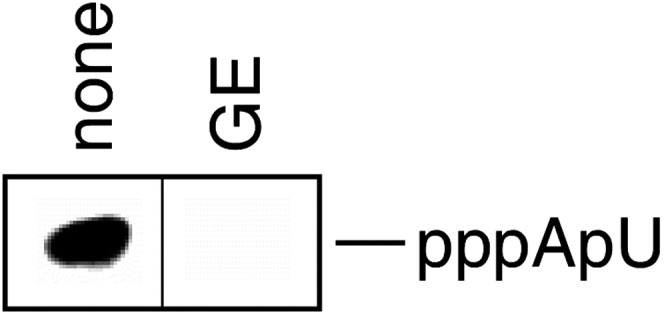
GE inhibits nucleotide addition in transcription initiation (*de novo* transcription initiation). **DOI:** http://dx.doi.org/10.7554/eLife.02450.004

**Figure 1—figure supplement 2.**
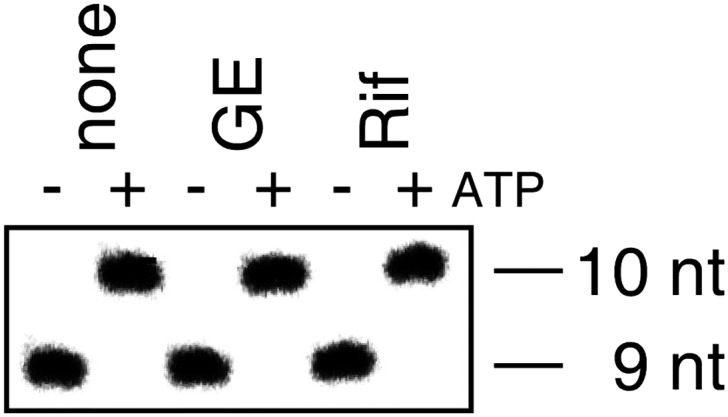
GE does not inhibit nucleotide addition in transcription elongation (reconstituted transcription elongation complexes). **DOI:** http://dx.doi.org/10.7554/eLife.02450.005

GE is a non-ribosomally-synthesized cyclic heptapeptide (Figure 1A; Marazzi et al., 2005). The stereochemistry at four chiral centers of GE has been defined based on acid hydrolysis and gas chromatography, but the stereochemistry at five other chiral centers has not been defined (Figure 1A; Marazzi et al., 2005). Analogs of GE having modifications of the dmaDap, dhGln, and Ama residues, have been prepared by semi-synthetic derivatization of GE (Mariani et al., 2005).

Here we report the target and mechanism of transcription inhibition by GE. In addition, we report a series of crystal structures—including the first crystal structure of a substrate complex for de novo transcription initiation by a multisubunit RNAP—that define the structural relationships between GE and RNAP, GE and promoter DNA, GE and NTPs, and GE and rifamycins.

Our results show that GE inhibits RNAP through a novel binding site and novel mechanism. GE inhibits RNAP by binding to a site—the ‘GE target’—that overlaps the RNAP active-center ‘i’ and ‘i+1’ sites and that includes coordinating ligands of the RNAP active-center catalytic Mg^2+^ ion, Mg^2+^(I). Binding of GE sterically precludes binding of initiating NTPs to the i site, i+1 site, and Mg^2+^(I), and thereby blocks transcription initiation. GE is the first identified example of a non-nucleoside RNAP inhibitor that functions through direct interaction with the core catalytic components of the RNAP active-center: the i site, i+1 site, and Mg^2+^(I).

Our results further show that the GE target has three features that make it an unusually attractive target—a ‘privileged target’—for antibacterial drug discovery involving RNAP. First, the GE target includes functionally critical residues of the RNAP active center that cannot be substituted without loss of RNAP activity, and, therefore, that cannot be substituted to yield resistant mutants. Accordingly, the target-based resistance spectrum for GE is unusually small. Second, the GE target does not overlap the rifamycin target (the target of the most important RNAP inhibitors in current clinical use in antibacterial therapy; Ho et al., 2009). Accordingly, GE exhibits no or negligible cross-resistance with rifamycins. Third, the GE target is immediately adjacent to the rifamycin target. Accordingly, it is possible to link GE to a rifamycin to construct a bipartite inhibitor that binds simultaneously to the GE target and the rifamycin target and, therefore, that is exceptionally potent and exceptionally refractory to target-based resistance.

## Results

### Mechanism of inhibition by GE: inhibition of first nucleotide addition in transcription initiation

To define the mechanism of transcription inhibition by GE, we assessed effects of GE on individual reaction steps in transcription initiation and transcription elongation. Figure 1B shows that GE does not inhibit steps in transcription initiation up to and including formation of a competitor-resistant RNAP-promoter open complex (RP_o_). We infer that GE does not inhibit promoter binding, loading of promoter DNA into the RNAP active-center cleft, or promoter unwinding.

The results in Figure 1C show that GE inhibits nucleotide addition in transcription initiation. GE inhibits both primer-dependent transcription initiation (Figure 1C), and de novo transcription initiation (Figure 1—figure supplement 1). In primer-dependent transcription initiation, GE inhibits the first nucleotide-addition step, inhibiting the synthesis of a 3-nt RNA product from a 2-nt RNA primer and an NTP. In de novo transcription initiation, GE inhibits the first nucleotide-addition step, inhibiting the synthesis of a 2-nt RNA product from initiating NTPs.

The results in Figure 1D show that GE does not inhibit nucleotide addition in transcription elongation. GE does not inhibit transcription elongation upon addition of NTPs to a halted elongation complex (Figure 1D), and GE does not inhibit single nucleotide addition upon addition of an NTP to an elongation complex reconstituted from RNAP and a synthetic nucleic acid scaffold (Figure 1—figure supplement 2).

We conclude that GE specifically inhibits nucleotide addition in transcription initiation. The observation that GE inhibits nucleotide addition in initiation but not in elongation suggests that GE functions through a binding site that is available in RP_o_ but that is not available in an elongation complex—for example, a binding site that overlaps the RNAP active-center i and i+1 nucleotide binding sites, or the path of the RNA product from the i and i+1 nucleotide binding sites, and that therefore would be unoccupied in RP_o_ but occupied by RNA in an elongation complex.

The mechanism of transcription inhibition of GE is reminiscent of, but differs from, the mechanism of transcription inhibition by rifampin (Rif) and other members of the rifamycin class. Like GE, Rif does not inhibit formation of RP_o_ (Figure 1B; McClure and Cech, 1978). Also like GE, Rif inhibits nucleotide addition in transcription initiation, but does not inhibit nucleotide addition in transcription elongation (Figure 1C,D; Sippel and Hartmann, 1968). However, in contrast to GE, Rif does not generally inhibit the first nucleotide-addition step in transcription initiation (Figure 1C; McClure and Cech, 1978). Rif generally only inhibits synthesis of >2–3-nt RNA products and does so by binding to a site along the path of RNA from the RNAP active-center and sterically blocking RNA extension (Campbell et al., 2001; Feklistov et al., 2008). The observation that GE inhibits synthesis of 2-nt RNA products, whereas Rif generally only inhibits synthesis of >2–3-nt RNA products, suggests that GE functions through a binding site located closer than the Rif binding site to the RNAP active-center.

The mechanism of transcription inhibition by GE also differs from the mechanisms of transcription inhibition by other previously characterized RNAP inhibitors. Sorangicin (Sor) functions through the same binding site on RNAP as Rif and inhibits synthesis only of >2–3-nt RNA products (Campbell et al., 2005). Myxopyronin (Myx), corallopyronin (Cor), ripostatin (Rip), and lipiarmycin (Lpm) inhibit formation of RP_o_ (Ho et al., 2009). Streptolydigin (Stl), CBR703 (CBR), and microcin J25 (MccJ25) inhibit nucleotide addition in both initiation and elongation (Artsimovitch et al., 2003; Mukhopadhyay et al., 2004; Ho et al., 2009). We conclude that GE inhibits transcription through a novel mechanism.

### Target of inhibition by GE: RNAP active-center i and i+1 sites

#### Isolation and characterization of GE-resistant mutants

**Figure 2.**
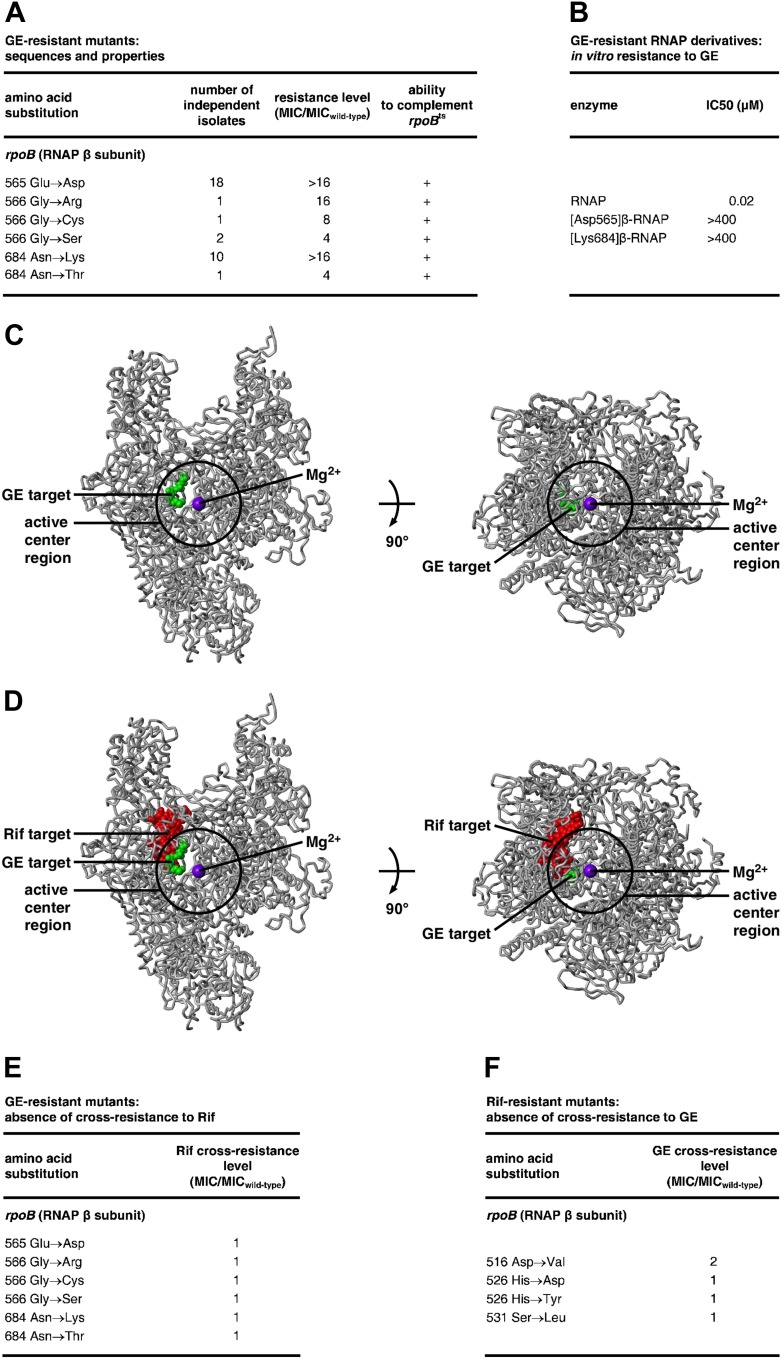
Target of transcription inhibition by GE: RNAP active-center i and i+1 sites. (**A**) GE^R^ mutants obtained following saturation mutagenesis of *E. coli rpoB* and *rpoC*. (**B**) GE^R^ phenotype of RNAP derivatives purified from GE^R^ mutants. (**C**) The GE target overlaps the RNAP active-center region. Structure of RNAP (gray ribbons; black circle for active-center region; violet sphere for Mg^2+^(I); β’ non-conserved region and σ omitted for clarity; Mukhopadhyay et al., 2008), showing sites of GE-resistant substitutions (green; sequences from A and Supplementary file 2C). Two orthogonal views. (**D**) The GE target does not overlap the Rif target. Structure of RNAP, showing sites of GE^R^ substitutions (green; sequences from A and Supplementary file 2C) and Rif^R^ substitutions (red; Jin and Gross, 1988; Severinov et al., 1993). (**E**) GE^R^ mutants are not cross-resistant to Rif. (**F**) Rif^R^ mutants are not cross-resistant to GE. See Figure 2—figure supplements 1, 2. **DOI:** http://dx.doi.org/10.7554/eLife.02450.006

To identify the target in RNAP for GE, we performed saturation mutagenesis of genes encoding *Escherichia coli* RNAP β and β′ subunits, and isolated and characterized mutants conferring GE-resistance (GE^R^). We performed saturation mutagenesis using a set of ‘doped’ oligonucleotide primers designed to introduce all possible nucleotide substitutions at all codons for all residues located within 30 Å of the RNAP active-center i and i+1 sites (primer sequences in Supplementary file 2A). We identified 33 independent single-substitution GE^R^ mutants (Figure 2A). All mapped to the RNAP β subunit (Figure 2A). The GE^R^ substitutions comprised six distinct substitutions at three sites in RNAP β: residues 565, 566, and 684 (Figure 2A). Minimal inhibitory concentration (MIC) assays indicate that all six GE^R^ substitutions result in at least moderate resistance (≥fourfold higher MIC) and that two result in high-level resistance (≥16-fold higher MIC; Figure 2A; Supplementary file 2B). Complementation assays indicate that each GE^R^ mutant is able to complement an *rpoB*^ts^ mutant for growth at the non-permissive temperature, indicating that each GE^R^ RNAP derivative is sufficiently functional in transcription to support viability (Figure 2A). RNAP purified from GE^R^ mutants exhibited resistance in vitro (Figure 2B), indicating that the GE^R^ phenotype at the cellular level is attributable to resistance at the enzymatic level. We conclude that RNAP is the functional cellular target for GE, and that RNAP β residues 565, 566, and 684 comprise a determinant essential for transcription inhibition by GE.

**Figure 2—figure supplement 1.**
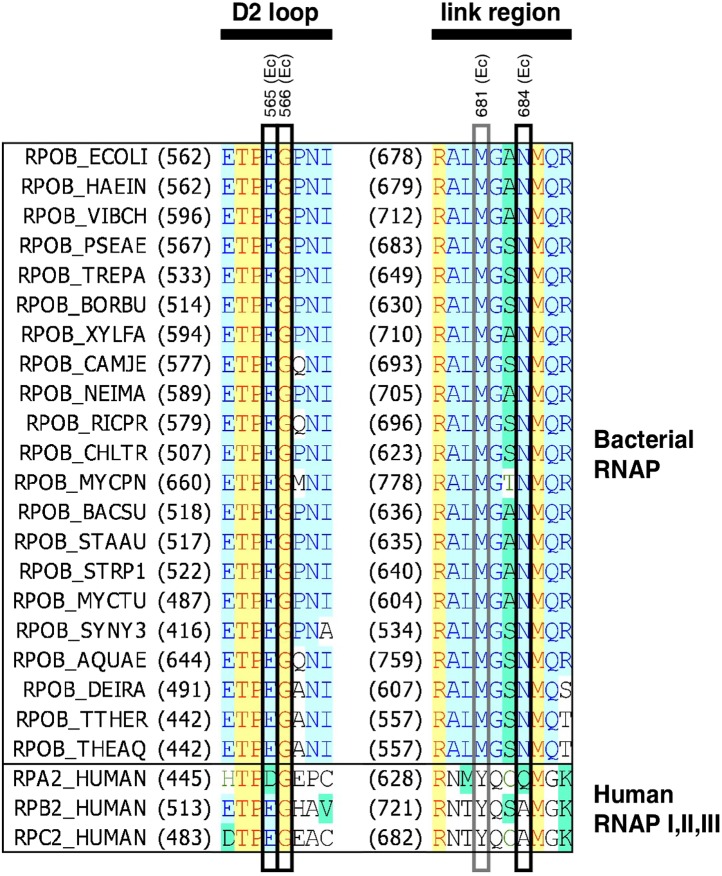
Location of GE target in sequence of RNAP β subunit. Sequence alignments for the β subunits of bacterial RNAP (top twenty-one sequences) and corresponding subunits of human RNAP I, RNAP II, and RNAP III (bottom three sequences), showing locations of GE^R^ substitutions in *E. coli* (black rectangles; sequences from Figure 2A) and *S. pyogenes* (black and gray rectangles; sequences in Supplementary file 2C). Species are as follows: *E. coli* (ECOLI), *Haemophilus influenzae* (HAEIN), *Vibrio cholerae* (VIBCH), *Pseudomonas aeruginosa* (PSEAE), *Treponema pallidum* (TREPA), *Borrelia burgdorferi* (BORBU), *Xylella fastidiosa* (XYLFA), *Campylobacter jejuni* (CAMJE), *Neisseria meningitidis* (NEIMA), *Rickettsia prowazekii* (RICPR), *Chlamydia trachomatis* (CHLTR), *Mycoplasma pneumoniae* (MYCPN), *Bacillus subtilis* (BACSU), *Staphylococcus aureus* (STAAU), *Streptococcus pyogenes* (STRP1), *Mycobacterium tuberculosis* (MYCTU), *Synechocystis sp.* PCC 6803 (SYNY3), *Aquifex aeolicus* (AQUAE), *Deinococcus radiodurans* (DEIRA), *Thermus thermophilus* (THETH), *Thermus aquaticus* (THEAQ), and *Homo sapiens* (HUMAN). **DOI:** http://dx.doi.org/10.7554/eLife.02450.007

**Figure 2—figure supplement 2.**
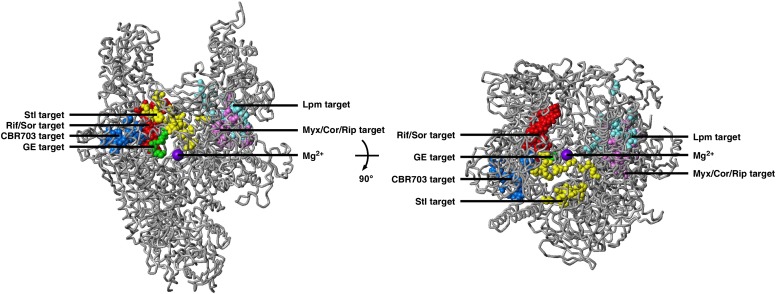
Relationship between GE target and targets of other RNAP inhibitors. The GE target does not overlap the targets of Rif, Sor, Stl, CBR703, Myx, and Lpm. Structure of bacterial RNAP (gray ribbons; violet sphere for active-center Mg^2+^; β' nonconserved region and σ omitted for clarity; Mukhopadhyay et al., 2008), showing GE target (green; Figure 2C) and targets of Rif and Sor (red; Ovchinnikov et al., 1981, 1983; Lisitsyn et al., 1984; Jin and Gross, 1988; Severinov et al., 1993, 1994; Garibyan et al., 2003, Campbell et al., 2005; Xu et al., 2005; Rodriguez-Verdugo et al., 2013; ES and RHE, unpublished), Stl (yellow; Lisitsyn et al., 1985; Heisler et al., 1993; Severinov et al., 1995; Tuske et al., 2005), CBR703 (blue; Artsimovitch et al., 2003; X Wang and RHE, unpublished), Myx (magenta; Mukhopadhyay et al., 2008), and Lpm (cyan; Ebright, 2005; Srivastava et al., 2011; RY Ebright, DD, and RHE, unpublished). Views as in Figure 2C. **DOI:** http://dx.doi.org/10.7554/eLife.02450.008

Analysis of a panel of *Streptococcus pyogenes* mutants carrying single-substitutions within the RNAP active-center region indicates that substitutions at residues corresponding to *E. coli* RNAP β residues 565, 681, and 684 confer a GE^R^ phenotype (Supplementary file 2C). We conclude that the region comprising RNAP β residues 565-566 and 681-684 constitutes a determinant essential for transcription inhibition by GE in both Gram-negative and Gram-positive bacterial RNAP.

The sites of GE^R^ substitutions are conserved in RNAP from both Gram-negative and Gram-positive bacteria (Figure 2—figure supplement 1). This is consistent with, and accounts for, the observation that GE inhibits RNAP from both Gram-negative and Gram-positive bacteria (Supplementary file 1B). Two sites of GE^R^ substitutions, β residues 681 and 684, are not conserved in human RNAP I, II, and III (Figure 2—figure supplement 1). This is consistent with, and accounts for, the observation that GE does not inhibit human RNAP I, II, and III (Supplementary file 1B; Ciciliato et al., 2004).

#### GE target

In the three-dimensional structure of RNAP, the sites of GE^R^ substitutions are located adjacent to each other and form a compact determinant (‘GE target’; Figure 2C). The GE target is located in the RNAP active-center region (Figure 2C). The GE target overlaps the RNAP active-center i and i+1 nucleotide binding sites, and comprises residues in two active-center subregions: the ‘D2 loop’ and the ‘link region’ (Figure 2—figure supplement 1). The RNAP active center contains two nucleotide binding sites—the i site and the i+1 site--flanking the catalytic Mg^2+^ ion, Mg^2+^(I) (Zhang and Landick, 2009). The i site serves as the binding site for the first initiating NTP in de novo transcription initiation, and as the binding site for the 3′-nucleotide of the RNA primer in primer-dependent transcription initiation and RNA product in transcription elongation. The i+1 site serves as the binding site for the second initiating NTP in de novo transcription initiation, and as the binding site for the extending NTP in primer-dependent transcription initiation and transcription elongation (Zhang and Landick, 2009). The D2 loop and the link region play roles in nucleotide addition, transcriptional fidelity, and transcriptional pausing (Libby et al., 1989; Landick et al., 1990; Toulokhonov et al., 2007; Weinzierl 2010, 2012; Gordon et al., 2012). The location of the GE target suggests that GE inhibits RNAP through direct interference with the function of the i and i+1 nucleotide binding sites and/or of Mg^2+^(I).

The GE target is located approximately midway between Mg^2+^(I) and the Rif target (Figure 2C,D). The location is consistent with the hypothesis of the preceding section that the inhibition of the first nucleotide-addition step by GE, but only of subsequent nucleotide-addition steps by Rif, is attributable to the closer proximity of the GE binding site to the RNAP active-center.

#### Relationship between GE target and targets of previously characterized RNAP inhibitors

The GE target is located adjacent to, but does not overlap, the Rif target (Figure 2D). Consistent with the absence of overlap, GE^R^ mutants do not exhibit cross-resistance with Rif (Figure 2E; Supplementary file 2D), and, conversely, Rif^R^ mutants do not exhibit cross-resistance with GE (Figure 2F; Supplementary file 2E; Ciciliato et al., 2004).

The GE target also does not overlap the targets of the previously characterized RNAP inhibitors Sor, Myx, Cor, Rip, Lpm, Stl, and CBR703 (Figure 2—figure supplement 2). Accordingly, GE^R^ mutants exhibit no cross-resistance with Sor, Myx, Cor, Rip, Lpm, Stl, and CBR703 (Supplementary file 2F).

#### Unusually small size of GE target

The GE target is strikingly small. The GE target comprises only six substitutions and only three sites in *E. coli* RNAP (Figure 2A), and has dimensions of just ∼16 Å × ∼10 Å × ∼9 Å (Figure 2C). The GE target is much smaller than the Rif target (71 substitutions and 27 sites; ∼30 Å × ∼25 Å × ∼10 Å; Figure 2D; Jin and Gross, 1988; Severinov et al., 1993). The GE target also is much smaller than the targets of other RNAP inhibitors, including the Myx/Cor/Rip target (28 substitutions and 19 sites; Figure 2—figure supplement 2; Mukhopadhyay et al., 2008), the Lpm target (30 substitutions and 20 sites; Figure 2—figure supplement 2; Srivastava et al., 2011; DD, and RHE, unpublished), the Stl target (27 substitutions and 19 sites; Figure 2—figure supplement 2; Tuske et al., 2005), the CBR703 target (23 substitutions and 13 sites; Figure 2—figure supplement 2; Artsimovitch et al., 2003; X Wang and RHE, unpublished), and the MccJ25 target (86 substitutions and 52 sites; Mukhopadhyay et al., 2004). The GE target also is small relative to the size of GE. We infer that the genetically defined GE target corresponds to just part of the GE binding site on RNAP, not the full GE binding site on RNAP (in contrast to the genetically defined targets of Rif and other previously characterized RNAP inhibitors, which correspond to full inhibitor binding sites; Ho et al., 2009). Specifically, we infer that the GE binding site comprises not only the residues at which GE^R^ substitutions are obtained, but also evolutionarily invariant, functionally essential, residues of the RNAP active center that cannot be substituted without loss of RNAP function, and thus cannot be substituted to confer GE-resistance. According to this hypothesis, the full GE binding site on RNAP includes not only the genetically-defined GE target, but also the full, or nearly the full, active-center i and i+1 sites; and GE bound to its target would be positioned to interfere directly, through steric clash, with function of the i and i+1 sites and/or Mg^2+^(I).

The unusually small size of the GE target-based resistance spectrum (six substitutions at three sites in *E. coli*; ∼1/10 the size of the target-based resistance spectrum for Rif, and ∼1/10 to ∼1/5 the sizes of the target-based resistance spectra for other RNAP inhibitors) has a potentially important practical implication. Namely, the frequency of spontaneous mutations yielding target-dependent GE-resistance is expected to be unusually small (∼1/10 to ∼1/5 the frequency of spontaneous mutations yielding target-dependent resistance to Rif and other RNAP inhibitors). In view of the fact that spontaneous mutations yielding target-dependent Rif-resistance are a major problem in antibacterial therapy with Rif (Ho et al., 2009), the smaller size of the GE target-based resistance spectrum is a potentially important advantage.

### Structural basis of inhibition by GE: crystal structure of RNAP-GE

#### GE binds to the GE target

**Figure 3.**
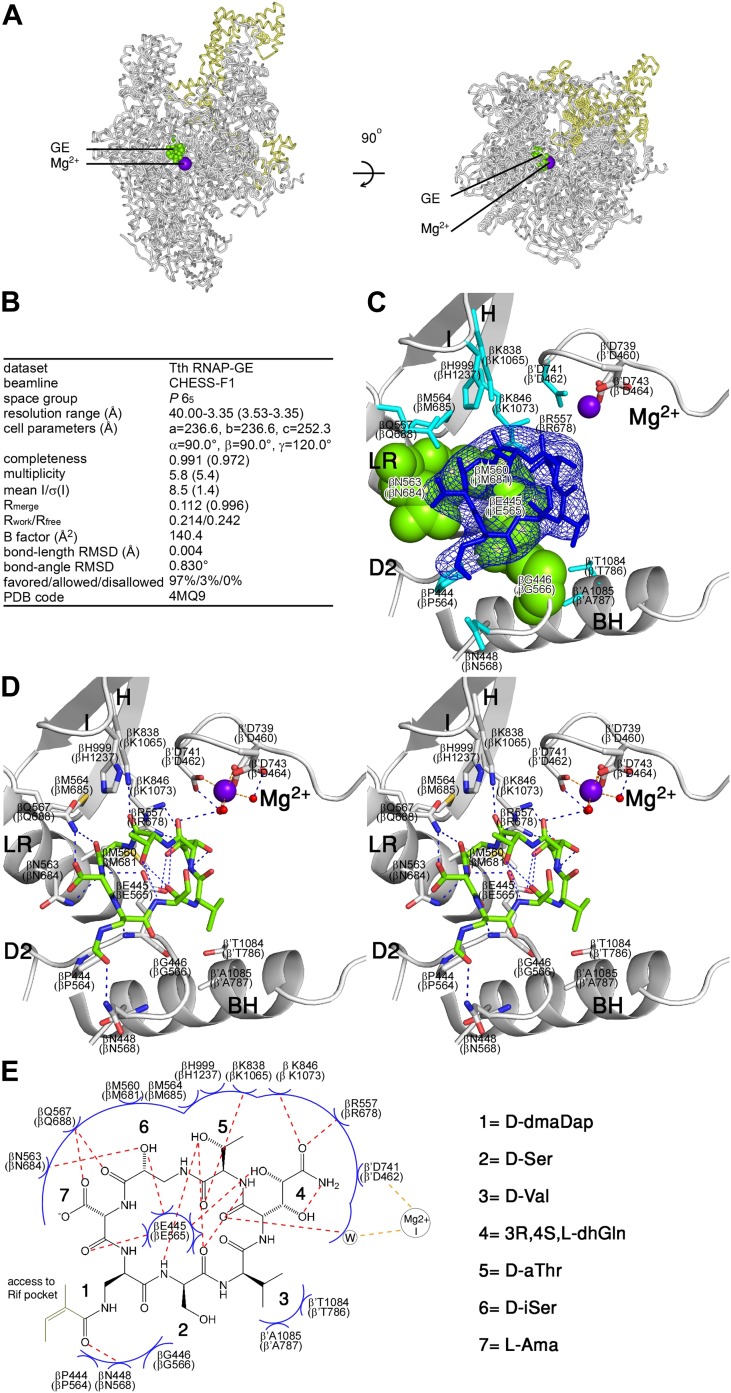
Structural basis of transcription inhibition by GE: crystal structure of RNAP-GE. (**A**) Overall structure. Green, GE; violet sphere, Mg^2+^(I); yellow, σ. (**B**) Crystallographic data and refinement statistics. (**C**) Electron density and atomic model for GE. Blue mesh, mF_o_-DF_c_ omit map for GE (contoured at 2.5σ); blue sticks, GE; gray ribbons, RNAP backbone; green surfaces, RNAP residues at which substitutions confer GE-resistance (Figure 2A; Supplementary file 2C); cyan sticks, additional RNAP residues that contact GE; gray and red sticks additional RNAP residues that coordinate Mg^2+^(I); violet sphere, Mg^2+^(I). D2, LR, H, I, Mg^2+^, and BH denote the RNAP D2 loop, link region, H region, I region, Mg^2+^ loop, and bridge helix. RNAP residues are numbered both as in *T. thermophilus* RNAP and as in *E. coli* RNAP (in parentheses). (**D**) Contacts between RNAP and GE (stereodiagram). Gray ribbons, RNAP backbone; gray sticks, RNAP carbon atoms; green, GE carbon atoms; red, oxygen atoms; blue, nitrogen atoms; red spheres, water molecules; violet sphere, Mg^2+^(I). Blue dashed lines, H-bonds; orange dashed lines, coordinate-covalent bonds. (**E**) Contacts between RNAP and GE (schematic). Red dashed lines, H-bonds; orange dashed lines, coordinate-covalent bonds; blue arcs, van der Waals interactions; W, water molecule. See Figure 3—figure supplement 1. **DOI:** http://dx.doi.org/10.7554/eLife.02450.009

To define the structural basis of transcription inhibition by GE, we determined a crystal structure of *Thermus thermophilus* RNAP holoenzyme in complex with GE at 3.35 Å resolution (Figure 3). Figure 3A shows that GE binds to the genetically-defined GE target, confirming the hypothesis that the GE target represents a determinant for binding of GE to RNAP. The structure shows that GE occupies the RNAP i and i+1 sites and makes direct interactions with the D2 loop, the link region, and an RNAP Asp residue and water molecule that coordinate Mg^2+^(I) (Figure 3B–E). The structure provides strong support to the hypothesis that GE inhibits RNAP by directly interfering with function of the i and i+1 sites and/or Mg^2+^(I).

**Figure 3—figure supplement 1.**
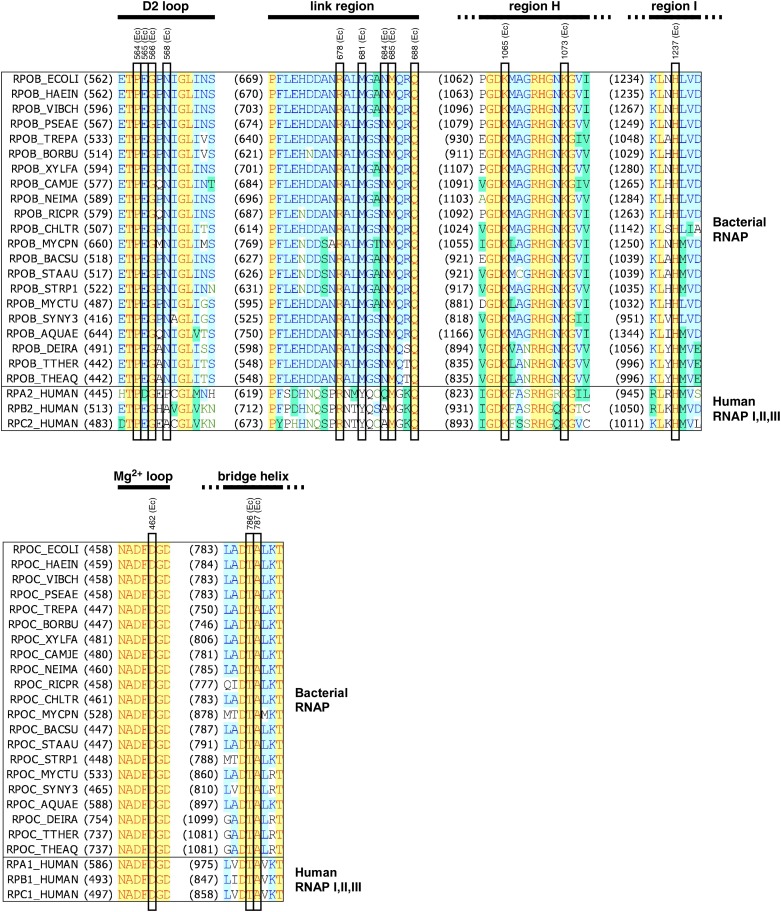
Structural basis of transcription inhibition by GE. Location of contacting residues in the sequences of RNAP β subunit (top) and RNAP β' subunit (bottom). Sequence alignments for the β and β' subunits of bacterial RNAP (top twenty-one sequences in each panel) and corresponding subunits of human RNAP I, RNAP II, and RNAP III (bottom three sequences in each panel), showing locations of residues that contact GE in the crystal structure of RNAP-GE (black rectangles; identities from Figure 3E), and locations of the RNAP D2 loop, link region, H region, I region, Mg^2+^ loop, and bridge helix (black bars; boundaries from Sweetser et al., 1987 and Weinzierl, 2010). Species names are as in Figure 2—figure supplement 2. **DOI:** http://dx.doi.org/10.7554/eLife.02450.010

#### RNAP residues at which GE-resistance substitutions occur contact GE

**Figure 4.**
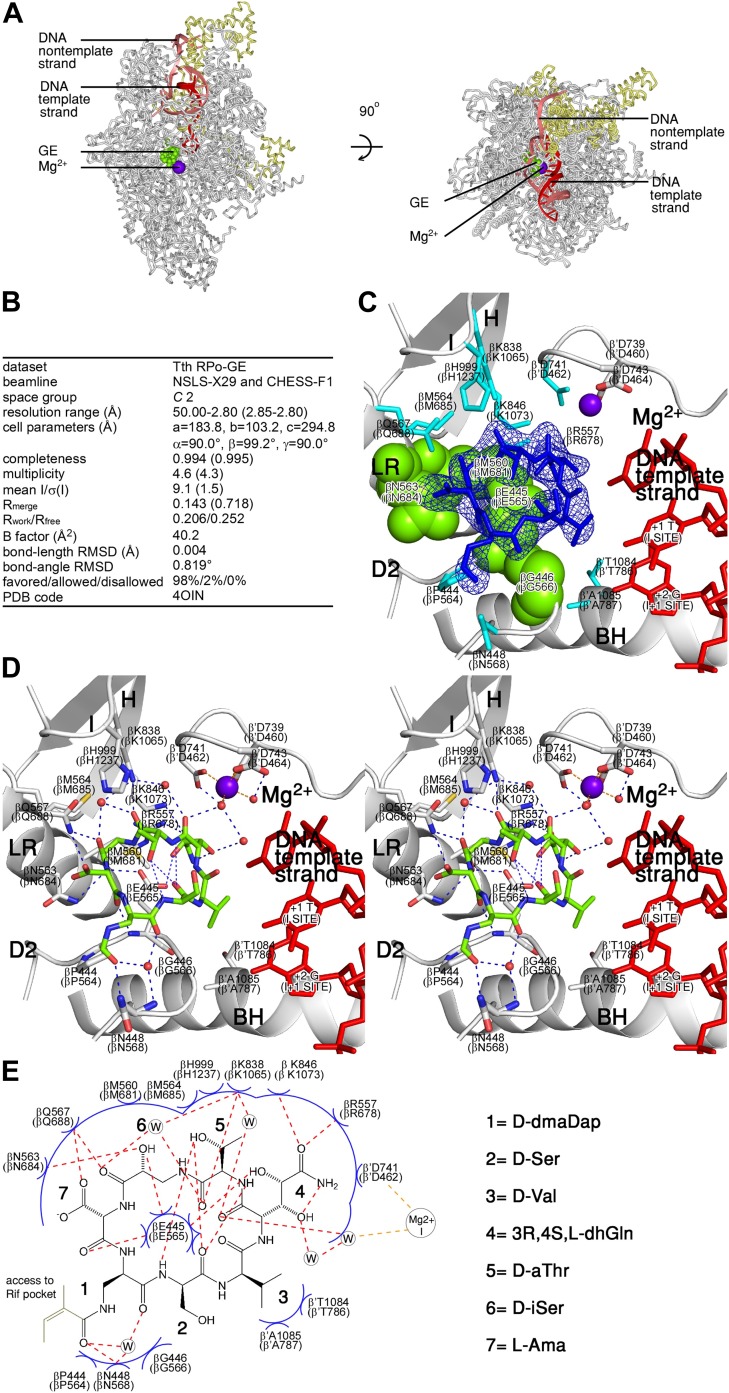
Structural basis of transcription inhibition by GE: crystal structure of RP_o_-GE. (**A**) Overall structure. (**B**) Crystallographic data and refinement statistics. (**C**) Electron density and atomic model for GE. (**D**) Contacts between RP_o_ and GE (stereodiagram). (**E**) Contacts between RP_o_ and GE (schematic). See Figure 4—figure supplements 1, 2. **DOI:** http://dx.doi.org/10.7554/eLife.02450.011

**Figure 5.**
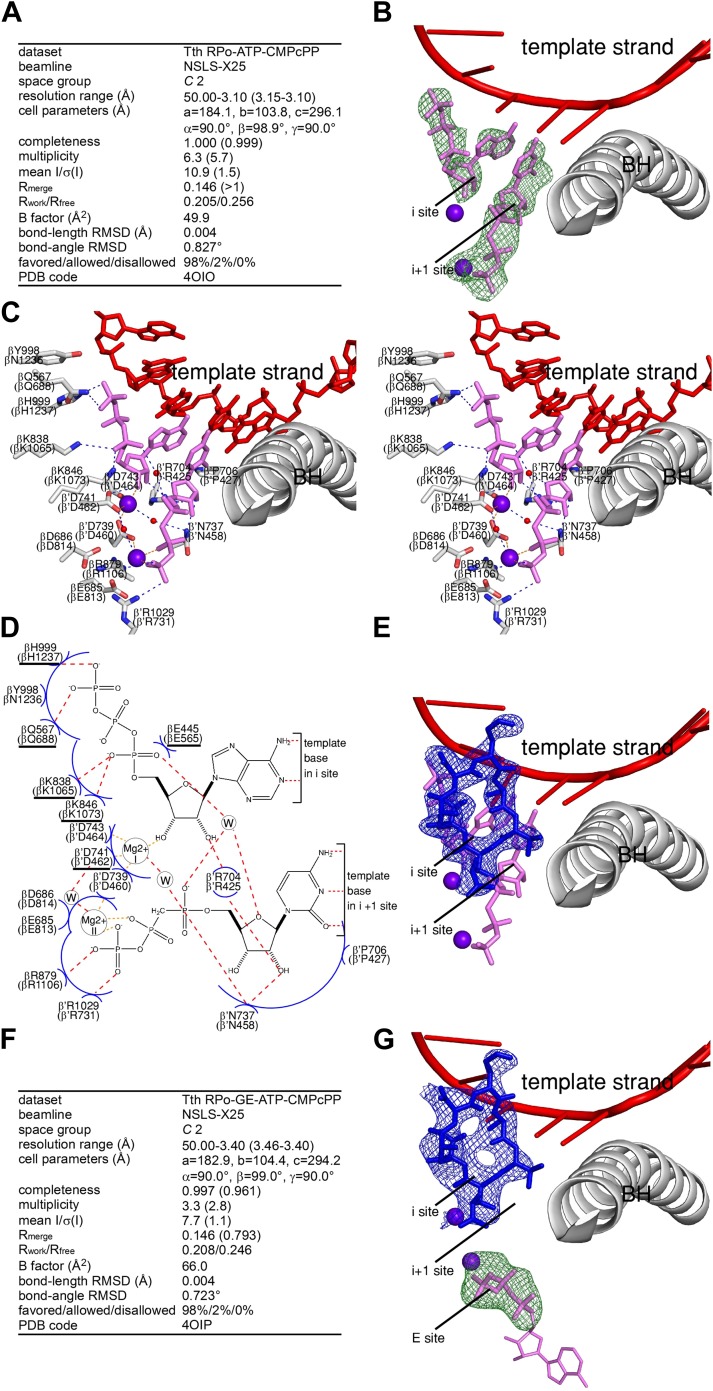
Relationship between GE and initiating NTPs: mutually exclusive binding. (**A**) Crystal structure of RP_o_-ATP-CMPcPP: crystallographic data and refinement statistics. (**B**) Crystal structure of RP_o_-ATP-CMPcPP: electron density and model. Green mesh, mF_o_-DF_c_ omit map for ATP and CMPcPP:Mg^2+^(II) (contoured at 2.5σ); pink sticks, ATP and CMPcPP; red ribbon, DNA template strand; gray ribbon, RNAP bridge helix; upper and lower violet spheres, Mg^2+^(I) and Mg^2+^(II). (**C**) Crystal structure of RP_o_-ATP-CMPcPP: contacts between RNAP and initiating NTPs (stereodiagram). Gray ribbon, RNAP bridge helix; gray sticks, RNAP carbon atoms; continuous red sticks, DNA atoms; pink sticks, ATP and CMPcPP carbon atoms; individual red sticks, oxygen atoms; individual blue sticks, nitrogen atoms; red spheres, water molecules; upper and lower violet spheres, Mg^2+^(I) and Mg^2+^(II). Blue dashed lines, H-bonds; orange dashed lines, coordinate-covalent bonds. (**D**) Crystal structure of RP_o_-ATP-CMPcPP: contacts between RNAP and initiating NTPs (schematic summary). Red dashed lines, H-bonds; orange dashed lines, coordinate-covalent bonds; blue arcs, van der Waals interactions; W, water molecule; underlined residues, GE-contacting residues in RP_o_-GE. (**E**) Superimposition of crystal structures of RP_o_-GE and RP_o_-ATP-CMPcPP: inferred steric clash between GE and initiating NTPs. Blue mesh, blue sticks, red sticks, and gray ribbon: mF_o_-DF_c_ omit map for GE, atomic model for GE, DNA template strand, and RNAP bridge helix from RP_o_-GE (Figure 4). Pink sticks and violet spheres: ATP, CMPcPP, Mg^2+^(I), and Mg^2+^(II) from RP_o_-ATP-CMPcPP. (**F**) Crystal structure of RP_o_-GE plus ATP and CMPcPP: crystallographic data and refinement statistics. (**G**) Crystal structure of RP_o_-GE plus ATP and CMPcPP: electron density and model. Blue mesh, mF_o_-DF_c_ omit map for GE (contoured at 2.7σ); blue sticks, GE; green mesh, mF_o_-DF_c_ omit map for NTP triphosphate:Mg^2+^(II) in RNAP E site (contoured at 2.7σ); pink sticks, NTP triphosphate; thin pink sticks, NTP sugar and base (projected); red ribbon, DNA template strand; gray ribbon, RNAP bridge helix; upper and lower violet spheres, Mg^2+^(I) and Mg^2+^(II). See Figure 5—figure supplement 1. **DOI:** http://dx.doi.org/10.7554/eLife.02450.015

All residues at which GE^R^ substitutions were obtained make direct contact with GE in the crystal structure: βGlu565, βGly566, βMet681, and βAsn684 (green in Figure 3C). (Here and elsewhere in the text, residues are numbered as in *E. coli* RNAP. In Figures 3–5, residues are numbered both as in *T. thermophilus* RNAP and as in *E. coli* RNAP.) The sidechain of βGlu565 penetrates the GE macrocycle and makes interactions with six of seven GE residues (Figure 3C–E). Substitution of βGlu565 is expected to disrupt multiple H-bonds and van der Waals interactions between RNAP and GE. Substitution of βGly566 by any residue other than Gly is expected to introduce steric clash between RNAP and GE. Substitution of βMet681 is expected to disrupt van der Waals interactions between RNAP and GE. Substitution of βAsn684 is expected to disrupt H-bonds and van der Waals interactions between RNAP and GE.

#### Additional RNAP residues contact GE

Besides the residues at which GE^R^ substitutions were obtained, 11 additional residues—all located in the RNAP active-center i and i+1 sites—make direct interactions with GE in the crystal structure: βPro564, βAsn568, βArg678, βMet685, βGln688, βLys1065, βLys1073, βHis1237, β′Asp462, β’Thr786, and β’Ala787 (cyan in Figure 3C). 10 of these additional residues are invariant in RNAP from bacteria to humans (Figure 3—figure supplement 1), and, for six, it is known that substitutions result in a loss of RNAP function (Kashlev et al., 1990; Mustaev et al., 1991; Sagitov et al., 1993; Sosunov et al., 2005; Jovanovic et al., 2011). We infer that these additional residues cannot be substituted without loss of RNAP function, and thus cannot be substituted to give rise to GE-resistance.

#### GE stereochemistry

The experimental electron density and inferred bonding patterns in the crystal structure define the stereochemistry at the five previously unassigned stereocenters of GE, as follows: D-dmaDap, D-Ser, D-Val, 3R,4S,L-dhGln, D-aThr, D-iSer, and L-Ama (Figure 3C–E). The assignment of stereochemistry at dhGln C3 is tentative. The assignments of stereochemistry at other stereocenters are firm.

#### RNAP-GE interactions

The crystal structure also defines the orientation of GE relative to RNAP and the interactions between GE and RNAP (Figure 3C–E). GE binds within a shallow bowl-like depression formed by the D2 loop, the link region, and the Mg^2+^ loop [Mg^2+^(I) and three RNAP Asp residues that coordinate Mg^2+^(I)], and the H and I regions (Figure 3C,D). GE is oriented relative to RNAP such that the GE dhGln residue is directed toward Mg^2+^(I) and the GE dmaDap residue is directed toward the Rif pocket (Figure 3C–E).

The GE dhGln residue participates in a network of interactions, including H-bonds with RNAP βGlu565, βArg678, and βLys1073, an H-bond with a water molecule in the first coordination shell of Mg^2+^(I), and van der Waals interactions with RNAP β′Asp462, which is one of the three RNAP Asp residues that coordinate Mg^2+^(I) (Figure 3D–E). The GE aThr residue makes an H-bond with RNAP βLys1065 and van der Waals interactions with βGlu565, βMet685, βLys1073, and βHis1237. The GE iSer residue makes H-bonds with RNAP βGlu565, βAsn684, and βGln688, and van der Waals interactions with βMet681. The GE Ama residue makes H-bonds with RNAP βGlu565 and βGln688, and van der Waals interactions with βAsn684. The GE dmaDap residue makes an H-bond with RNAP βAsn568 and van der Waals interactions with βGly566 and βPro564. Atoms of the GE dmaDap sidechain distal to the sidechain carbonyl are disordered in the structure, indicating that these atoms exhibit static or dynamic conformational heterogeneity, and suggesting that these atoms make few or no interactions with RNAP (omitted in Figure 3C–D; gray in Figure 3E). The GE Ser residue makes van der Waals interactions with RNAP βGlu565. The GE Val residue makes van der Waals interactions with RNAP βGlu565, β′Thr786, and β’Ala787.

The structure accounts for the structure-activity relationships obtained from analysis of semi-synthetic derivatives of GE. Modification of the GE dhGln sidechain eliminates RNAP-inhibitory activity (Mariani et al., 2005), consistent with the participation by this sidechain in multiple H-bonds and van der Waals interactions with RNAP. Removal of the Ama sidechain reduces RNAP-inhibitory activity (Mariani et al., 2005), consistent with the participation of this sidechain in an H-bond and van der Waals interactions with RNAP. Substitutions of the dmaDap sidechain acyl moiety, including substitutions with bulky groups, has little or no effect on RNAP-inhibitory activity (Mariani et al., 2005), consistent with the fact that atoms of the acyl group are disordered in the structure and are inferred to make few or no interactions with RNAP.

### Structural basis of inhibition by GE: crystal structure of RP_o_-GE

To define effects of GE on interactions of RNAP with promoter DNA, we determined a crystal structure of RP_o_ in complex with GE at 2.8 Å resolution (Figure 4). The higher resolution of this structure (2.8 Å vs 3.35 Å) enables confirmation of the inferred stereochemical assignments at stereocenters of GE (Figure 4D,E) and enables identification of additional water-mediated H-bonds, including additional water-mediated H-bonds in the network of water-mediated interactions connecting GE to Mg^2+^(I) (Figure 4D–E; Figure 4—figure supplement 1).

**Figure 4—figure supplement 1.**
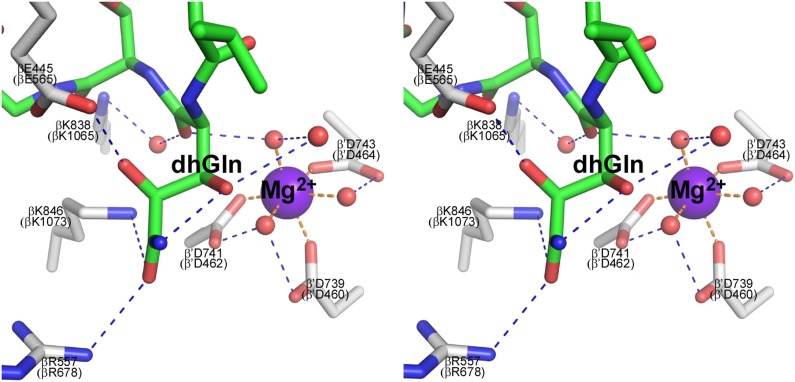
Structural basis of transcription inhibition by GE. Network of contacts to GE dhGln residue. Stereoview. Gray, RNAP carbon atoms. Green, GE carbon atoms. Red, oxygen atoms. Blue, nitrogen atoms. Violet sphere, Mg^2+^(I). Red spheres, water molecules. Dashed blue lines, H-bonds. Dashed orange lines, coordinate-covalent bonds. **DOI:** http://dx.doi.org/10.7554/eLife.02450.012

**Figure 4—figure supplement 2.**
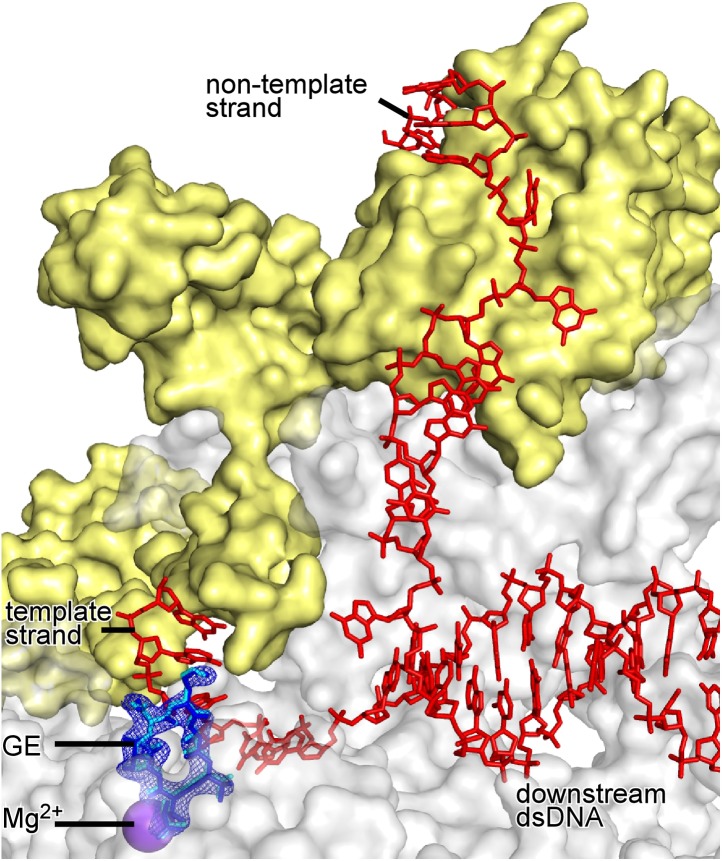
Absence of effects of DNA on GE conformation and RNAP-GE interactions. Superimposition of crystal structures of RP_o_-GE (Figure 4) and RNAP-GE (Figure 3). Blue mesh, blue sticks, red sticks, gray surface, yellow surface, and violet sphere: mF_o_-DF_c_ omit map for GE, atomic model for GE, DNA, RNAP, σ, and Mg^2+^(I) from crystal structure of RP_o_-GE (Figure 4). Cyan sticks, atomic model for GE from crystal structure of RNAP-GE (Figure 3). **DOI:** http://dx.doi.org/10.7554/eLife.02450.013

**Figure 4—figure supplement 3.**
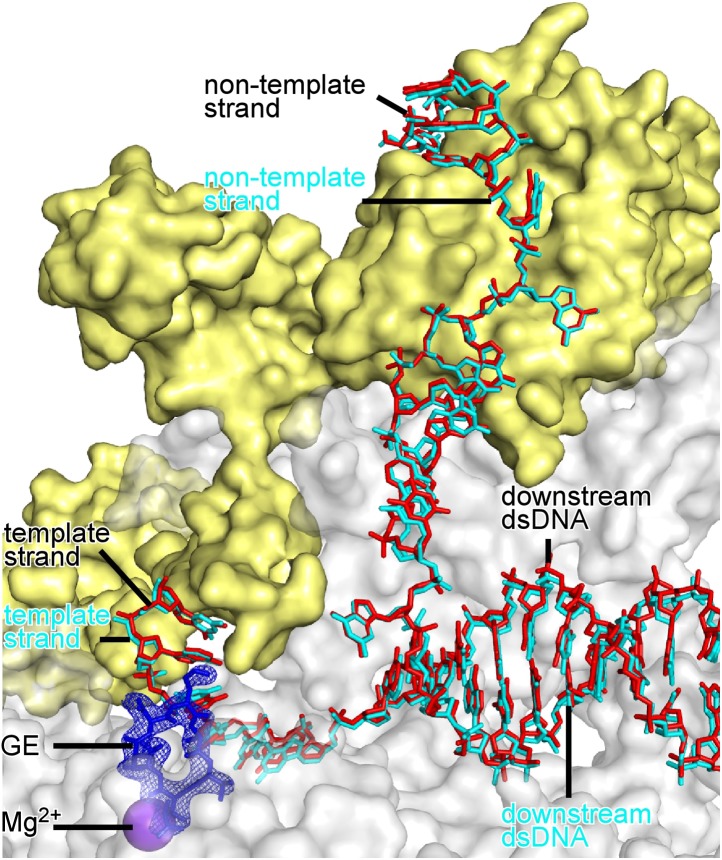
Absence of effects of GE on DNA conformation and RNAP-DNA interactions. Superimposition of crystal structures of RP_o_-GE (Figure 4) and RP_o_ (Zhang et al., 2012). Blue mesh, blue sticks, red sticks, gray surface, yellow surface, and violet sphere: mF_o_-DF_c_ omit map for GE, atomic model for GE, DNA, RNAP, σ, and Mg^2+^(I) from crystal structure of RP_o_-GE (Figure 4). Cyan sticks, DNA from crystal structure of RP_o_ (Zhang et al., 2012). **DOI:** http://dx.doi.org/10.7554/eLife.02450.014

Comparison of the structures of RNAP-GE (Figure 3) and RP_o_-GE (Figure 4) shows that promoter DNA binding to form RP_o_ does not change the conformation of GE or the interactions between RNAP and GE (Figure 4—figure supplement 2). Comparison of structures of RP_o_ (Zhang et al., 2012) and RP_o_-GE (Figure 4) show that GE does not change the conformation of DNA or the interactions between RNAP and DNA (Figure 4—figure supplement 3). The results provide a graphic confirmation of the results from Figure 1, indicating that GE does not inhibit formation of RP_o_ and, instead, inhibits a subsequent reaction required for the first nucleotide addition in transcription initiation.

### Relationship between GE and initiating nucleotides: mutually exclusive binding

#### Structural modelling of steric clash between GE and initiating nucleotides

As a first step to assess whether occupancy of the RNAP i and i+1 sites by GE interferes with the binding of nucleotides to the i and i+1 sites, we constructed a structural model of a primer-dependent transcription initiation complex by superimposing crystal structures of RP_o_-GE (Figure 4), RP_o_ in complex with a 2-nt RNA primer occupying the i-1 and i sites (Zhang et al., 2012), and a transcription elongation complex containing an NTP in the i+1 site (Vassylyev et al., 2007) (Figure 5—figure supplement 1). The resulting structural model predicts severe steric clash between GE and both the RNA 3′ nucleotide in the i site and the NTP in the i+1 site (Figure 5—figure supplement 1). The phosphate and base of the RNA 3′ nucleotide are predicted to clash with the aThr residue and Ama residue, respectively, of GE. The α-phosphate and base of the NTP in the i+1 site are predicted to clash with the dhGln residue and Val residue, respectively, of GE. The structural model strongly suggests that GE interferes with binding of nucleotides to the RNAP i and i+1 sites.

#### Crystal structure defining interactions between RP_o_ and initiating nucleotides in the absence of GE

As a second step to assess whether occupancy of the RNAP i and i+1 sites by GE interferes with the binding of nucleotides to the i and i+1 sites, and, in order to define how the triphosphate of the first initiating NTP interacts with RNAP and how interactions may be impacted by GE, we determined a crystal structure of RP_o_ in complex with initiating NTPs at 3.1 Å resolution (Figure 5A–D). To determine the structure, we soaked a pre-formed crystal of RP_o_ with the first initiating NTP (ATP) and a non-reactive analog of the second initiating NTP (CMPcPP). The electron density map shows unambiguous electron density for ATP in the i site and for CMPcPP:Mg^2+^(II) in the i+1 site (Figure 5B). The resulting structure provides the first structural information of a substrate complex for de novo transcription by a multi-subunit RNAP.

**Figure 5—figure supplement 1.**
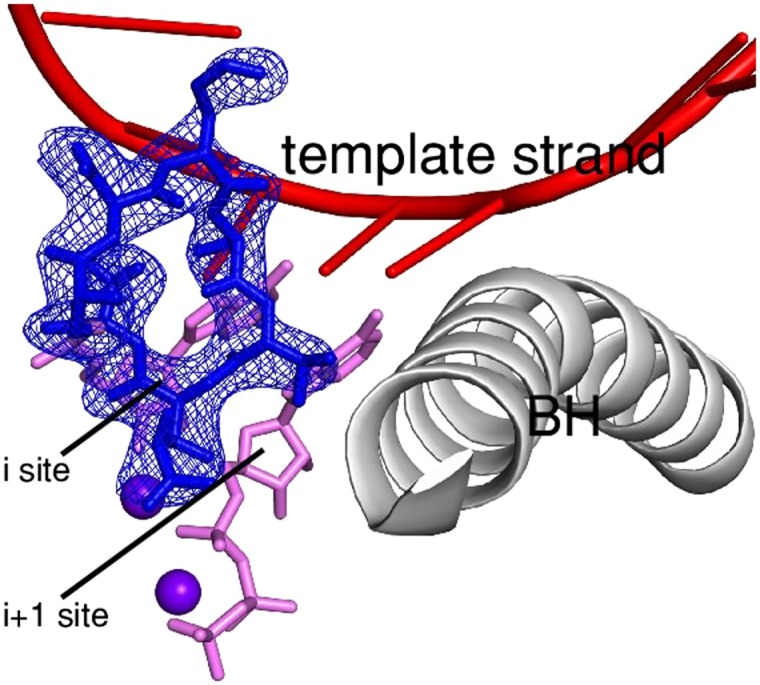
Relationship between GE and initiating NTPs. Superimposition of crystal structures of RP_o_-GE, RP_o_-GpA, and the transcription elongation complex: predicted steric clash between GE and NTPs in i and i+1 sites. Blue mesh, blue sticks, red sticks, gray ribbon: mF_o_-DF_c_ omit map for GE, atomic model for GE, DNA template strand, and RNAP bridge helix from crystal structure of RP_o_-GE (Figure 4). Upper pink sticks and violet sphere: RNA 3' nucleotide in i site and Mg^2+^(I) from crystal structure of RP_o_-GpA (Zhang et al., 2012). Lower pink sticks and violet sphere: NTP in i+1 site and Mg^2+^(II) from crystal structure of the transcription elongation complex (Vassylyev et al., 2007). **DOI:** http://dx.doi.org/10.7554/eLife.02450.016

The base and sugar moieties of the first initiating NTP make the same interactions with DNA and RNAP that the RNA 3′-nucleotide base and sugar make in a transcription elongation complex (Vassylyev et al., 2007; Figure 5C,D). The triphosphate of the first initiating NTP extends into the space that is occupied by the RNA-1 nucleotide in a transcription elongation complex, and makes H-bonds and salt-bridges through its γ-phosphate with RNAP βGln688 and βHis1237, and through its α-phosphate with RNAP βLys1065 and βLys1073 (Figure 5C,D). The observed interactions of the γ-phosphate and α-phosphate with βHis1237 and βLys1065 are consistent with, and account for, crosslinking results (Mustaev et al., 1991).

The base moiety of the second initiating NTP makes the same interactions with DNA and RNAP that the extending NTP base makes in an elongation complex (Vassylyev et al., 2007; Figure 5C,D). The sugar and triphosphate of the second initiating NTP make interactions characteristic of a ‘preinsertion-mode’ elongation complex, in which the sugar and triphosphate make only a subset of the interactions required for catalysis, and, in particular, in which the triphosphate approaches, but does not coordinate, Mg^2+^(I) (Vassylyev et al., 2007; Zhang and Landick, 2009; Martinez-Rucobo and Cramer, 2013; Figure 5C,D). The RNAP active center is not fully dehydrated and contains two ordered water molecules in the interface between the first and second initiating NTPs (red spheres in Figure 5C; ‘W’ in Figure 5D), consistent with expectation for a ‘preinsertion-mode’ complex. The RNAP trigger loop, which can adopt open or closed conformational states, adopts an open conformational state in this structure, further consistent with expectation for a ‘preinsertion-mode’ complex. It is believed that the ‘preinsertion-mode’ elongation complex is an obligatory functional intermediate in formation of the catalytically competent ‘insertion-mode’ elongation complex (Vassylyev et al., 2007; Zhang and Landick, 2009; Martinez-Rucobo and Cramer, 2013). We suggest, by analogy, that the ‘preinsertion-mode’ initiation complex defined herein is an obligatory functional intermediate in formation of the catalytically competent ‘insertion-mode’ initiation complex.

The determination of a crystal structure of a substrate complex for de novo initiation (Figure 5A–D) provided a firm foundation for structural modelling of relationships between GE and initiating nucleotides in a transcription initiation complex. Accordingly, we constructed a structural model by superimposing crystal structures of RP_o_-GE (Figure 4) and RP_o_-ATP-CMPcPP (Figure 5A–D). The resulting structural model shows severe steric clash between GE and both the first initiating NTP in the i site and the second initiating NTP in the i+1 site (Figure 5E). The structural model confirms the steric clashes predicted in the structural model built using an elongation complex structure (Figure 5—figure supplement 1), and reveals new, particularly severe, steric clashes involving the triphosphate of the first initiating NTP (Figure 5E). The steric clashes with the triphosphate entail essentially complete steric interpenetration of the triphosphate α, β, and γ phosphates with the GE aThr and Ama residues. The structural model very strongly suggests that GE interferes with binding of nucleotides to the RNAP i and i+1 sites.

#### Crystal structure defining interactions between RP_o_ and initiating nucleotides in the presence of GE

To test directly whether GE interferes with binding of initiating NTPs to the i and i+1 sites, we compared NTP occupancies of the i and i+1 sites in the absence of GE to those in the presence of GE. To do this, we compared electron density maps for crystals of RP_o_ soaked with ATP and CMPcPP (Figure 5A,B) to electron density maps for crystals of RP_o_ first soaked with GE and then soaked with ATP and CMPcPP (Figure 5F,G). As described above, electron density maps obtained by soaking a crystal of RP_o_ with ATP and CMPcPP show unambiguous electron density for ATP and CMPcPP in the i and i+1 sites (Figure 5B). In contrast, electron density maps obtained by soaking a crystal of RP_o_ first with GE, and then with ATP and CMPcPP show unambiguous electron density for GE, but show no density for ATP or CMPcPP in the i and i+1 sites (Figure 5G). Instead, electron density attributable to an NTP triphosphate is seen in a region adjacent to the i+1 site termed the ‘E site’ (Figure 5G). The E site previously has been reported as a binding site for a non-complementary NTP and has been proposed to serve as an entry site for NTPs on the pathway of NTP binding (Westover et al., 2004). The pair of structures indicating that initiating NTPs occupy the i and i+1 sites in the absence of GE (Figure 5B), but do not occupy the i and i+1 sites in the presence of GE (Figure 5G), show graphically that GE interferes with binding of initiating NTPs to the i and i+1 sites.

In further work, we performed analogous crystal-soaking experiments to assess effects of GE on occupancy of 3′-deoxy-3′-amino-ATP and CTP (a non-reactive analog of the first initiating NTP and a reactive second initiating NTP) and of ATP and CTP (a reactive first initiating NTP and a reactive second initiating NTP). In these cases, soaking of nucleotides into RP_o_ in the absence of GE yielded, respectively, an ‘insertion mode’ substrate complex with nucleotides in the i and i+1 sites, and a product complex with a 2-nt RNA product (to be published elsewhere). In contrast, in each case, soaking nucleotides into RP_o_ pre-soaked with GE yielded a complex with electron density for GE, no electron density for nucleotides in the i and i+1 sites, and density attributable to an NTP triphosphate in the E site.

We conclude that GE interferes with binding of initiating nucleotides to the RNAP i and i+1 sites.

### Relationship between GE and Rif: simultaneous binding

#### Partial-competitive binding of GE and Rif

The observation that the GE target is adjacent to the Rif target (Figure 2D) raises the possibility that binding of GE to RNAP may affect binding of Rif to RNAP. As a first step to assess interactions between GE and Rif, we performed fluorescence-detected binding experiments (Feklistov et al., 2008) monitoring RNAP-Rif interaction in the absence and presence of GE.

**Figure 6.**
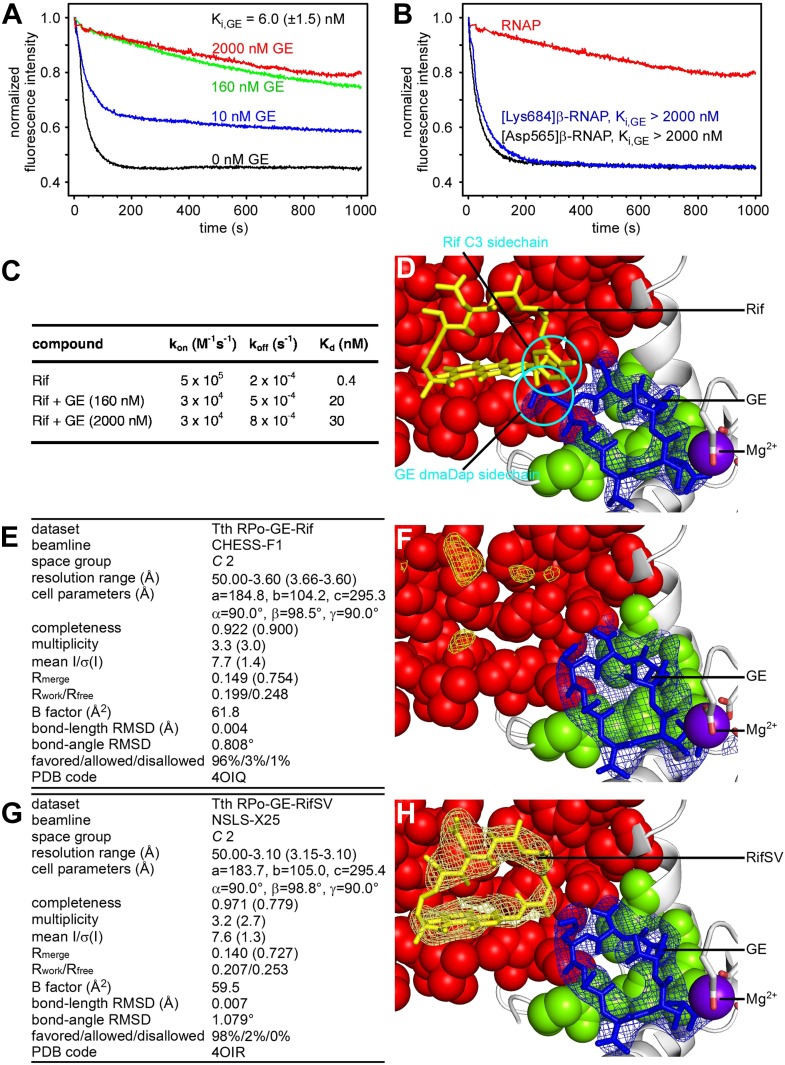
Relationship between GE and Rif: simultaneous binding. (**A**) Partial-competitive binding of GE and Rif: association kinetics for Rif in presence of 0–2000 nM GE. (**B**) Partial-competitive binding of GE and Rif: association kinetics for Rif in presence of 2000 nM GE, using wild-type RNAP (red) and GE-resistant RNAP derivatives [Asp565]β-RNAP (black) and [Lys684]β-RNAP (blue). (**C**) Partial-competitive binding of GE and Rif: k_on_, k_off_, and K_d_ for Rif in absence of GE and in presence of saturating GE (160 nM or 2000 nM; ∼30 × K_i_ or ∼300 × K_i_). (**D**) Superimposition of crystal structures of RP_o_-GE and RNAP-Rif: inferred simultaneous binding. Blue mesh, blue sticks, gray ribbon, and violet sphere: mF_o_-DF_c_ omit map for GE, atomic model for GE, RNAP, and Mg^2+^(I) from RP_o_-GE (Figure 4). Yellow sticks: Rif from RNAP-Rif (PDB: 1YNN). Green surfaces, GE target residues at which substitutions confer GE-resistance; red surfaces, residues at which substitutions confer Rif-resistance. (**E**) Crystal structure of RP_o_-GE plus Rif: crystallographic data and refinement statistics. (**F**) Crystal structure of RP_o_-GE plus Rif: electron density and model. Yellow mesh, patchy electron density potentially attributable to Rif (mF_o_-DF_c_ omit map; contoured at 2.7σ). Other colors as in **D**. (**G**) Crystal structure of RP_o_-GE-RifSV: crystallographic data and refinement statistics. (**H**) Crystal structure of RP_o_-GE-RifSV: electron density and model. Yellow mesh, mF_o_-F_c_ omit map for RifSV (contoured at 2.7σ); yellow sticks, RifSV. Other colors as in **D**. See Figure 6—figure supplements 1, 2. **DOI:** http://dx.doi.org/10.7554/eLife.02450.017

The results in Figure 6A–C show that GE inhibits the binding of Rif to RNAP. GE decreases k_on_ for Rif ∼20-fold, increases k_off_ for Rif ∼fourfold, and increases the equilibrium dissociation constant (K_d_) for Rif ∼80-fold (Figure 6C). The equilibrium dissociation constant for inhibition of RNAP-Rif interaction by GE (K_i_) is 6 nM, which is comparable to the IC50 for inhibition of RNAP by GE (Figure 6A; Figure 6—figure supplement 1; Supplementary file 1B). GE^R^ RNAP derivatives do not exhibit inhibition of RNAP-Rif interaction by GE, indicating that the inhibition requires specific interactions of GE with the GE target (Figure 6B).

**Figure 6—figure supplement 1.**
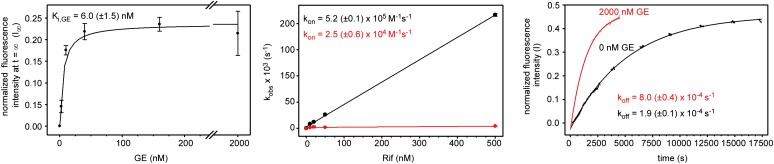
Relationship between GE and Rif: effects of GE on RNAP-Rif interaction. Left panel, association kinetics: I_∞_ for RNAP-Rif interaction in the presence of 0, 2.5, 10, 40, 160, or 2000 nM GE. Center panel, association kinetics: k_obs_ for RNAP-Rif interaction in the presence of 0 or 2000 nM GE. Right panel, dissociation kinetics: I for RNAP-Rif interaction in the presence of 0 or 2000 nM GE. **DOI:** http://dx.doi.org/10.7554/eLife.02450.018

**Figure 6—figure supplement 2.**
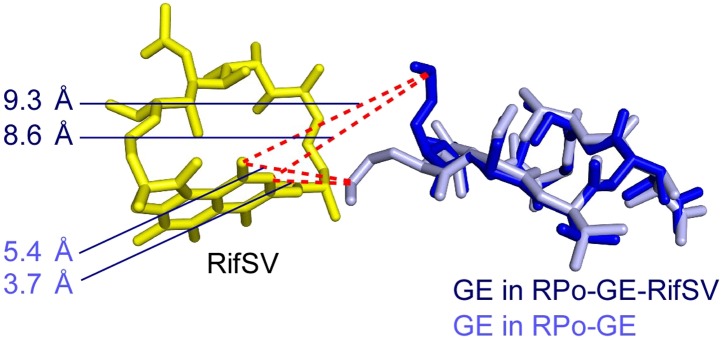
Relationship between GE and Rif: superimposition of crystal structures of RP_o_-GE and RP_o_-GE-RifSV showing differences in conformations of GE dmaDap residue. Blue sticks, yellow sticks, and blue numbers: GE, RifSV, and distances between GE dmaDap sidechain carbonyl carbon and RifSV C3 and O^4^ atoms, in RP_o_-GE-RifSV. Gray sticks and gray numbers: GE in RP_o_-GE and corresponding distances calculated for the GE conformation in RP_o_-GE. **DOI:** http://dx.doi.org/10.7554/eLife.02450.019

However, the results in Figure 6A–C also show that GE does not preclude the binding of Rif to RNAP. Thus, even at saturating concentrations of GE, RNAP-Rif interaction still occurs (Figure 6A) and still exhibits a submicromolar K_d_ (K_d_ = 30 nM; Figure 6C).

The results quantitatively fit a model of partial competitive binding—i.e., a model in which X inhibits the binding of Y, but in which X and Y can bind simultaneously at sufficient concentrations; Segel, 1975. We infer that GE inhibits the binding of Rif to RNAP, but that GE and Rif can bind simultaneously to RNAP at sufficient concentrations. The observation that GE inhibits the binding of Rif is consistent with the fact that the GE target is adjacent to the Rif target (Figure 2D), enabling steric clash between GE bound to the GE target and Rif bound to the Rif target. The observation that GE does not preclude the binding of Rif is consistent with the observation that the GE target does not overlap with the Rif target (Figure 2D–F).

#### Structural modelling of simultaneous binding of GE and a rifamycin

As a next step to assess interactions between GE and Rif, we constructed a structural model of GE bound to the GE target and Rif bound to the Rif target. To construct the model, we superimposed the crystal structure of RP_o_-GE (Figure 4) on a crystal structure of RNAP-Rif (Campbell et al., 2005). The structural model predicts that GE bound to the GE target is located immediately adjacent to Rif bound to the Rif target (Figure 6D). The structural model further predicts that there is steric clash between GE bound to the GE target and Rif bound to the Rif target, but that clash is limited to the dmaDap sidechain of GE and the C3 atom and sidechain of Rif (cyan in Figure 6D). The predicted adjacent binding and steric clash are consistent with the observation that GE and Rif compete for binding (Figure 6A–C). The predicted limitation of the steric clash to a single moiety of GE and a single moiety of Rif is consistent with the observation that GE and Rif can bind simultaneously to RNAP at sufficient concentrations (Figure 6A–C).

#### Crystal structures defining simultaneous binding of GE and a rifamycin

As a next step to assess interactions between GE and rifamycins, we sought to determine crystal structures of RP_o_ bound simultaneously to GE and a rifamycin. In a first effort, we soaked crystals of RP_o_ with GE and Rif (Figure 6E,F). The resulting electron density maps showed unambiguous electron density for GE in the GE target, but only limited density in the Rif target (Figure 6F). In a second effort, noting that steric clash may be limited to the dmaDap sidechain of GE and the C3 atom and sidechain of Rif (Figure 6D), we soaked crystals of RP_o_ with GE and rifamycin SV (RifSV), a Rif analog that lacks the C3 sidechain and that retains high RNAP-inhibitory and antibacterial potency (Figure 6G–H; Sensi et al., 1966). In this case, the resulting electron density maps showed unambiguous electron density for GE in the GE target and for RifSV in the Rif target (Figure 6H). Occupancy levels for both GE and RifSV were 1, indicating that GE and RifSV were bound simultaneously to RNAP in the crystal. The inability to obtain a structure with simultaneously bound ligands upon crystal soaking with GE and Rif, but ability to obtain a structure with simultaneously bound ligands upon crystal soaking with GE and RifSV, highlights the contribution of the rifamycin C3 region to steric clash between GE and rifamycins.

The conformation of the GE dmaDap residue differs in RP_o_-GE and RP_o_-GE-RifSV (Figure 6—figure supplement 2). The GE dmaDap sidechain in RP_o_-GE-RifSV is rotated by ∼110°, in a direction that increases the distance between the dmaDap sidechain carbonyl carbon and the RifSV C3 atom from 3.7 Å to 8.6 Å and thereby alleviates steric clash. This observation highlights the contribution of the GE dmaDap residue to steric clash between GE and rifamycins.

### Bipartite inhibitors: GE-rifamycin and GE-sorangicin

#### Structural modelling of GE-rifamycin and GE-sorangicin bipartite inhibitors

**Figure 7.**
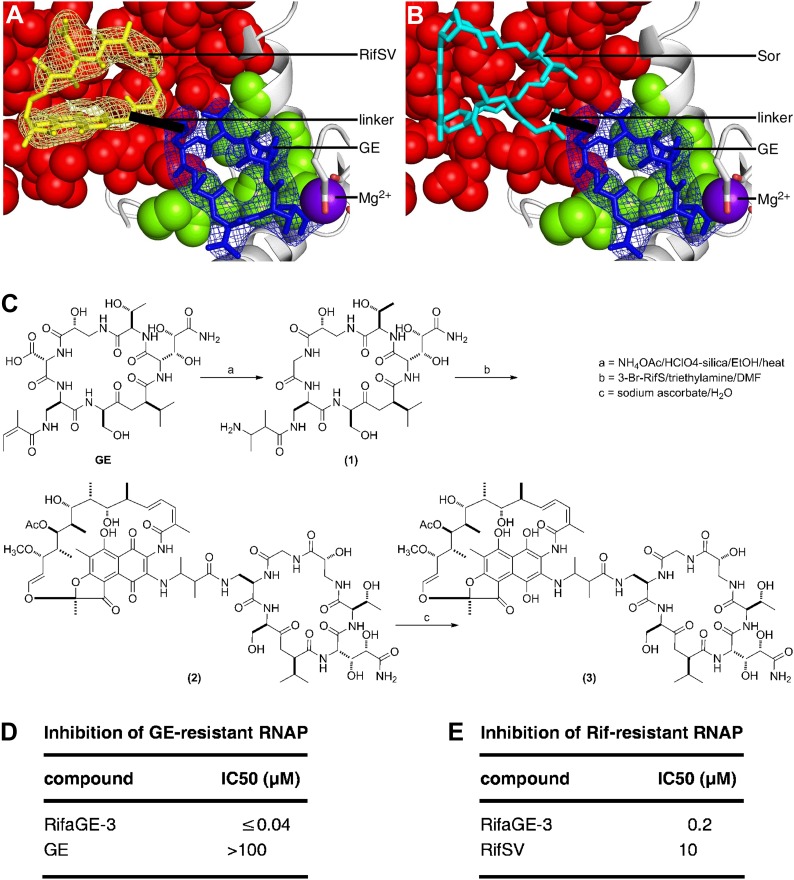
Bipartite inhibitors: GE-rifamycin and GE-sorangicin. (**A**) Proposed bipartite inhibitor having GE dmaDap sidechain linked to RifSV C3 or O^4^ atom. Crystal structure of RP_o_-GE-RifSV. Black line, linker. Other colors as in Figure 6H. (**B**) Proposed bipartite inhibitor having GE dmaDap sidechain linked to Sor sidechain carboxyl. Superimposition of crystal structures of RPo-GE and RNAP-Sor (PDB: 1YNJ). Cyan, Sor; red, residues at which substitutions confer Sor-resistance. Other colors as in **A**. (**C**) Synthesis of bipartite inhibitor having GE dmaDap sidechain linked to RifSV C3 atom (RifaGE-3). (**D**) Inhibition of GE-resistant RNAP ([Asp565]β-RNAP) by RifaGE-3. (**E**) Inhibition of Rif-resistant RNAP ([Asn516]β-RNAP) by RifaGE-3. **DOI:** http://dx.doi.org/10.7554/eLife.02450.020

The crystal structure of RP_o_-GE-RifSV immediately suggests the possibility of constructing a bipartite compound comprising GE, linked through its dmaDap residue, to a rifamycin, linked through its C3 or O^4^ atom (Figure 7A). Fortuitously, the GE dmaDap residue is one of three GE residues that have chemical reactivity that can be, and has been, exploited for derivatization by semi-synthesis (sole α,β-unsaturated amide moiety in GE; enables site-selective hydrolysis, ozonolysis, and 1,4-addition; Mariani et al., 2005; YWE and RHE, unpublished), and the rifamycin C3 and O^4^ atoms have chemical reactivities that can be, and extensively have been, exploited for derivatization of rifamycins by semi-synthesis (Sensi et al., 1966). Still more fortuitously, the GE dmaDap residue and the rifamycin C3 and O^4^ atoms are positions that can be modified without loss of activity (Sensi et al., 1966; Mariani et al., 2005). Accordingly, synthesis of such a bipartite compound not only is possible, but also is tractable. Such a bipartite compound is expected to be able to bind simultaneously to the GE target (through the GE moiety) and the Rif target (through the rifamycin moiety). Accordingly, such a compound is expected to have exceptionally high binding affinity, exceptionally high RNAP-inhibitory potency, and an ability to overcome resistance arising from substitutions in one of the GE target and the Rif target.

Sor, a compound not structurally-related to rifamycins, functions by binding to the Rif binding site (Campbell et al., 2005; Ho et al., 2009). Structural modelling of RP_o_ having GE bound to the GE target and Sor bound to the Rif binding site (Figure 7B), indicates that GE and Sor, like GE and a rifamycin, may be able to bind simultaneously to RNAP, and may be able to be linked to yield a bipartite inhibitor with exceptionally high binding affinity, exceptionally high RNAP-inhibitory potency, and an ability to overcome resistance arising from substitutions in one of the GE target and the Sor target. Fortuitously, the part of Sor that is predicted to be closest to, and potentially linkable, to GE is the Sor carboxyl moiety (Figure 7B), which has chemical reactivity that can be, and has been, exploited for derivatization of Sor by semi-synthesis, and which can be modified without loss of RNAP-inhibitory activity and antibacterial activity (Jansen et al., 1990).

#### Synthesis and evaluation of a GE-rifamycin bipartite inhibitor

We have synthesized and evaluated a bipartite inhibitor comprising a GE derivative and RifSV, covalently connected through the GE-derivative dmaDap sidechain, the RifSV C3 atom, and a one-atom linker (‘RifaGE-3’; compound 3 of Figure 7C). To prepare the bipartite inhibitor, we employed a three-step procedure involving: (a) site-selective introduction of an amino group into the GE dmaDap sidechain through 1,4-addition (with concomitant heat/acid-catalyzed decarboxylation of the GE Ama sidechain), (b) reaction with 3-bromo-rifamycin S, and (c) reduction with sodium ascorbate (Figure 7C). The resulting bipartite inhibitor inhibits wild-type RNAP with an IC50 at the limit of detection of the assay (IC50 ≤40 nM), inhibits GE^R^ RNAP >2500-fold more potently than GE (Figure 7D), and inhibits Rif^R^ RNAP 50-fold more potently than RifSV (Figure 7E). The biochemical, microbiological, and structural characterization of the bipartite inhibitor, as well as the optimization of linkage sites, linker lengths, and synthetic methods for preparation of bipartite inhibitors, will be reported separately. Nevertheless, the results in Figure 7C–E provide proof-of-concept for the synthesis, the high potency against wild-type RNAP, and the ability to overcome resistance of a GE-rifamycin bipartite inhibitor.

## Discussion

Our results establish GE inhibits RNAP through a novel mechanism and a novel target. Our results show that GE inhibits the first nucleotide-addition step in transcription initiation (Figure 1), show that GE functions through a binding site that overlaps the RNAP active-center i and i+1 sites (Figure 2), define the structural basis of RNAP-GE interaction and RP_o_-GE interaction (Figures 3,4), and show that GE prevents binding of initiating NTPs to the RNAP i and i+1 sites (Figure 5).

Our results further establish that the binding site on RNAP for GE is adjacent to, but does not substantially overlap, the binding site on RNAP for the rifamycin antibacterial drugs (Figure 2D–F), show that GE and a rifamycin can bind simultaneously to their adjacent binding sites in RNAP (Figure 6), and show that GE and a rifamycin can be covalently linked, through the GE dmaDap sidechain and the rifamycin C3-O^4^ region, to yield a bipartite RNAP inhibitor that binds to both the GE target and the rifamycin target (Figure 7).

Three features of the GE target, identified in this work, indicate that the GE target is an unusually attractive target—a ‘privileged target’—for antibacterial drug discovery involving RNAP. First, since most residues of the GE binding site are functionally critical residues of the RNAP active center that cannot be substituted without loss of RNAP activity, the target-based resistance spectra of an antibacterial compound that functions through the GE binding site will be small (∼1/10 the size of the target-based resistance spectrum of Rif; ∼1/10 to ∼1/5 the size of the target-based resistance spectra of RNAP inhibitors; Figure 2D; Figure 2—figure supplement 2). Second, since the GE binding site is different from the rifamycin binding site, an antibacterial compound that functions through the GE binding site will not exhibit target-based cross-resistance with rifamycins (Figure 2E,F; Supplementary file 2D,E). Third, since the GE binding site is adjacent to, but does not substantially overlap, the rifamycin binding site (Figures 2D and 6), an antibacterial compound that functions through the GE binding site can be linked to a rifamycin or a sorangicin to construct a bipartite, bivalent inhibitor that binds to both the GE target and the rifamycin target and, therefore, that is exceptionally potent and exceptionally refractory to target-based resistance (Figure 7).

## Materials and methods

### RNAP inhibitors

GE23077 (GE) was prepared from cultures of *Actinomadura* sp. DSMZ 13491 as in Ciciliato et al. (2004). Sorangicin (Sor) was prepared from cultures of *Sorangium cellulosum* strain So cel2 as in Irschik et al. (1987). Lipiarmycin (Lpm) was prepared from cultures of *Actinoplanes deccanensis* as in Coronelli et al. (1975). (±)E,E-myxopyronin B (Myx) was synthesized as in Ebright and Ebright (2013). Rifampin (Rif), rifamycin SV (RifSV), streptolydigin (Stl), and CBR703 were purchased from Sigma–Aldrich (St. Louis, MO), Sigma–Aldrich, Sourcon-Padena (Tübingen, Germany), and Maybridge (Tintagel, UK), respectively.

### Plasmids

Plasmid pRL706 encodes C-terminally hexahistidine-tagged *E. coli* RNAP β subunit under control of the *trc* promoter (Severinov et al., 1997). Plasmid pRL663 encodes C-terminally hexahistidine-tagged *E. coli* RNAP β′ subunit under control of the *tac* promoter (Wang et al., 1995). Plasmid pKD46 carries a temperature-sensitive replication origin, confers ampicillin-resistance, and encodes λ Exo, Beta, and Gam, under control of the *P*_*araB*_ promoter (Datsenko and Wanner, 2000). Plasmid pAKE604 confers kanamycin-resistance and sucrose-sensitivity (El-Sayed et al., 2001).

### *E. coli* RNAP holoenzyme

*E. coli* RNAP, [Asn516]β-RNAP, [Asp565]β-RNAP, and [Lys684]β-RNAP core and holoenzyme were prepared from *E. coli* strain XE54 (Tang et al., 1994) transformed with pRL706, pRL706-516N, pRL706-565D, and pRL706-684K, respectively, using procedures essentially as in Niu et al. (1996). *E. coli* RNAP holoenzyme derivatives site-specifically labelled with fluorescein at σ^70^ residue 517 ([F^517^]σ^70^-RNAP holoenzyme derivatives) were prepared as in Knight et al. (2005).

### *T. thermophilus* RNAP holoenzyme

*T. thermophilus* RNAP holoenzyme was prepared as in Zhang et al. (2012).

### RNAP-inhibitory activity

Fluorescence-detected RNAP-inhibition assays were performed by a modification of the procedure of Kuhlman et al. (2004). Reaction mixtures contained (20 μl): 0–100 μM test compound, bacterial RNAP holoenzyme (75 nM *E. coli* RNAP holoenzyme or *E. coli* RNAP holoenzyme derivative, 75 nM *Staphylococcus aureus* RNAP core enzyme and 300 nM *S. aureus* σ^A^ [prepared as in Srivastava et al. 2011], 75 nM *Mycobacterium tuberculosis* RNAP core enzyme and 300 nM *M. tuberculosis* σ^A^ [prepared as in Srivastava et al. 2011], or 75 nM *T. thermophilus* RNAP holoenzyme), 20 nM DNA fragment containing the bacteriophage T4 N25 promoter (positions −72 to +367; prepared by PCR from plasmid pARTaqN25-340-tR2 [Liu, 2007]), 100 μM ATP, 100 μM GTP, 100 μM UTP, and 100 μM CTP, in TB (50 mM Tris–HCl, pH 8.0, 100 mM KCl, 10 mM MgCl_2_, 1 mM DTT, 10 μg/ml bovine serum albumin, 5% methanol, and 5.5% glycerol). Reaction components other than DNA and NTPs were pre-incubated 10 min at 37°C. Reactions were carried out by addition of DNA and incubation 15 min at 37°C, followed by addition of NTPs and incubation 60 min at 37°C. DNA was removed by addition of 1 μl 5 mM CaCl_2_ and 2 U DNase I (Ambion, Grand Island, NY), followed by incubation 90 min at 37°C. RNA was quantified by addition of 100 μl Quant-iT RiboGreen RNA Reagent (Life Technologies, Grand Island, NY; 1:500 dilution in 10 mM Tris–HCl, pH 8.0, 1 mM EDTA), followed by incubation 10 min at 22°C, followed by measurement of fluorescence intensity (excitation wavelength = 485 nm and emission wavelength = 535 nm; GENios Pro microplate reader [Tecan, Männedorf, Switzerland]).

Radiochemical assays with human RNAP I/II/III were performed essentially as in Sawadogo and Roeder (1985). Reaction mixtures contained (20 µl): 0–100 µM GE, 8 U HeLaScribe Nuclear Extract (Promega, Madison, WI), 1 µg human placental DNA (Sigma–Aldrich), 400 μM ATP, 400 μM [α^32^P]UTP (0.11 Bq/fmol), 400 μM CTP, 400 μM GTP, 50 mM Tris–HCl, pH 8.0, 7 mM HEPES-NaOH, 70 mM (NH_4_)_2_SO_4_, 50 mM KCl, 12 mM MgCl_2_, 5 mM DTT, 0.1 mM EDTA, 0.08 mM phenylmethylsulfonyl fluoride, and 16% glycerol. Reaction components other than DNA and NTPs were pre-incubated 10 min at 30°C, DNA was added and reaction mixtures were incubated 15 min at 30°C, NTPs were added and reaction mixtures were incubated 60 min at 30°C. Reaction mixtures were spotted on DE81 filter discs (Whatman, Kent, UK; pre-wetted with water) and incubated 1 min at room temperature. Filters were washed with 3 × 3 ml Na_2_HPO_4_, 2 × 3 ml water, and 3 ml ethanol, using a filter manifold (Hoefer, Holliston, MA). Filters were placed in scintillation vials containing 10 ml Scintiverse BD Cocktail (Thermo Fisher, Waltham, MA), and radioactivity was quantified by scintillation counting (LS6500; Beckman–Coulter, Brea, CA).

Half-maximal inhibitory concentrations (IC50s) were calculated by non-linear regression in SigmaPlot (SPSS, Chicago, IL).

### Antibacterial activity

Minimum inhibitory concentrations (MICs) were quantified using broth microdilution assays (Clinical and Laboratory Standards Institute, 2009), using a starting cell density of 3 × 10^4^ cfu/ml, LB broth (Sambrook and Russell, 2001), and an air atmosphere for *E. coli* D21f2tolC (*tolC:*Tn*10 rfa lac28 proA23 trp30 his51 rpsL173 ampC tsx81*; strain with cell-envelope defects resulting in increased susceptibility to hydrophobic agents, including GE; Fralick and Burns-Keliher, 1994; unpublished data), and using a starting cell density of 3 × 10^4^ cfu/ml, Bacto Todd Hewitt broth (TH broth; BD Biosciences, San Jose, CA), and a 7% CO_2_/6% O_2_/4% H_2_/83% N_2_ atmosphere for *S. pyogenes* and *M. catarrhalis*.

### GE-resistant mutants: isolation and sequencing

Saturation mutagenesis of *rpoB* plasmid pRL706 and *rpoC* plasmid pRL663 was performed by use of PCR amplification with ‘doped’ oligodeoxyribonucleotide primers (methods as in Mukhopadhyay et al. 2008). ‘Doped’ oligodeoxyribonucleotide primers corresponding to codons 136-143, 504-511, 512-522, 523-534, 535-541, 542-549, 563-573, 677-690, 758-763, 813-814, 829-835, 1054-1060, 1064-1074, 1102-1108, and 1233-1242 of the *rpoB* gene of plasmid pRL706, and codons 347-355, 425-429, 456-465, 779-792, and 934-943 of the *rpoC* gene of plasmid pRL663, were synthesized on an Applied Biosystems 392/394 automated DNA/RNA synthesizer (Foster City, CA) using solid-phase β-cyanoethylphosphoramidite chemistry (sequences in Supplementary file 2A). The level of ‘doping’ (nucleotide misincorporation) was selected to yield an average of 0.4–1 substitution(s) per molecule of oligodeoxyribonucleotide primer (equations in Hermes et al., 1989, 1990). Thus, the nucleotides corresponding to codons 758-763 and 813-814 of *rpoB*, and codons 425-429 of *rpoC* were synthesized using phosphoramidite reservoirs containing 92% of the correct phosphoramidite and 8% of a 1:1:1:1 mix of dA, dC, dG, and dT phosphoramidites (i.e., 94% total correct phosphoramidite and 6% total incorrect phosphoramidite). The nucleotides corresponding to codons 136-143, 504-511, 512-522, 523-534, 535-541, 542-549, 563-573, 677-690, 829-835, 1054-1060, 1064-1074, 1102-1108, and 1233-1242 of *rpoB*, and codons 347-355, 456-465, 779-792, and 934-943 of *rpoC* were synthesized using phosphoramidite reservoirs containing 98% of the correct phosphoramidite and 2% of a 1:1:1:1 mix of dA, dC, dG, and dT phosphoramidites, (i.e., 98.5% total correct phosphoramidite and 1.5% total incorrect phosphoramidite.) All other nucleotides were synthesized using phosphoramidite reservoirs containing 100% of the correct phosphoramidite. Mutagenesis reactions were performed using the QuikChange XL Site-Directed Mutagenesis Kit (Agilent/Stratagene, La Jolla, CA) with a “doped” oligodeoxyribonucleotide primer, a complementary oligodeoxyribonucleotide primer, and pRL706 or pRL663 as template (primers at 75-150 nM; all other components at concentrations as specified by the manufacturer). Mutagenized plasmid DNA was introduced by transformation into *E. coli* XL1-Blue (Agilent/Stratagene). Transformants (10^3^-10^4^ cells) were applied to LB-agar plates (Sambrook and Russell, 2001) containing 200 μg/ml ampicillin, plates were incubated 16 hr at 37°C, and plasmid DNA was prepared from the pooled resulting colonies. The resulting passaged mutagenized plasmid libraries for the 15 “doped” oligonucleotide primers targeting *rpoB* were pooled on an equimolar basis, and the resulting passaged mutagenized plasmid libraries for the five “doped” oligonucleotide primers targeting *rpoC* were pooled on an equimolar basis. Pooled, passaged mutagenized plasmid libraries for each gene were introduced by transformation into *E. coli* D21f2tolC. Transformants (∼10^3^ cells) were applied to LB-agar plates containing 200-500 μg/ml GE, 200 μg/ml ampicillin, and 1 mM IPTG; and plates were incubated 24-48 hr at 37°C. GE-resistant mutants were identified by the ability to form colonies on this medium, were confirmed by re-streaking on the same medium, were further confirmed by quantifying resistance levels in liquid cultures and accepting only isolates with >2-fold resistance (procedures as described below), and were demonstrated to contain plasmid-linked GE-resistant mutations by preparing plasmid DNA, transforming *E. coli* D21f2tolC with plasmid DNA, and plating transformants on the same medium. For each confirmed mutant, nucleotide sequences of *rpoB* and *rpoC* were determined by Sanger sequencing (eight primers per gene).

### GE-resistant mutants: complementation assays

Temperature-sensitive *E. coli* strain RL585 [*rpoB*^*am*^*cI supD*^*ts*^*43,74* Δ*(recA-srl)306 lacZ*^*am*^*2110 galEK*^*am*^ *leu*^*am*^ *trp*^*am*^ *sueA rpsL tsx srl301*::Tn*10-84*; Landick et al., 1990] was transformed with pRL706 or a pRL706 derivative, transformants (10^3^-10^4^ cells) were applied to LB-agar plates containing 200 μg/ml ampicillin, 1 mM IPTG, and 10 μg/ml tetracycline, plates were incubated 22 hr at 43°C, and bacterial growth was scored.

### GE-resistant mutants: transfer to chromosome

GE-resistant and Rif-resistant mutations were transferred from pRL706 derivatives to the chromosome of *E. coli* D21f2tolC by λ-Red-mediated recombineering (procedures analogous to those in Datsenko and Wanner 2000 and Sawitzke et al., 2007; but using chemical transformation rather than electroporation). DNA fragments (143 bp or 306 bp) containing *rpoB* segments with GE-resistant or Rif-resistant mutations were prepared by PCR amplification using pRL706 derivatives carrying GE-resistant and Rif-resistant mutations as templates and 5’-CAGGTGGTATCCGTCGGTGCGTCCCTG-3’ and 5’-CGTTCCATACCAGTACCAACCAGCGGC-3’ (for GE-resistant mutations) or 5′-GGATATGATCAACGCCAAGCCGATTTCCGCAGC-3′ and 5′-CGATACGGAGTCTCAAGGAAGCCGTATTCG-3′ (for Rif-resistant mutations) as primers. DNA fragments were purified by isolation by electrophoresis on 0.8% agarose (procedures as in Sambrook and Russell 2001) and extracted from gel slices using a Gel/PCR DNA Fragments Extraction Kit (IBI Scientific, Peosta, IA; procedures as specified by the manufacturer).

DNA fragments and co-selection/counter-selection plasmid pAKE604 (10 ng and 100 ng; for GE-resistant mutations) or DNA fragments only (30 ng; for Rif-resistant mutations) were introduced by transformation into chemically competent cells of *E. coli* D21f2tolC pKD46 (prepared by culturing *E. coli* D21f2tolC pKD46 in LB broth containing 200 μg/ml ampicillin and 1 mM arabinose at 30°C until OD = 0.6, pelleting cells, re-suspending cells in 85% LB, 10% PEG 3350, 5% DMSO, and 50 mM MgCl_2_, and flash freezing in dry-ice/ethanol), and transformants were cultured 3.5 hr at 37°C with shaking, applied to LB-agar plates containing 500 μg/ml GE and 40 μg/ml kanamycin (for GE-resistant mutations) or 1–2 μg/ml Rif (for Rif-resistant mutations), and incubated 24-30 hr at 37°C. Isolates containing chromosomal GE-resistant or Rif-resistant mutations were identified by the ability to form colonies on media containing GE or Rif, were confirmed by re-streaking on the same media, and were verified to have lost temperature-sensitive plasmid pKD46 by re-streaking on LB-agar plates containing 0 or 200 μg/ml ampicillin. For GE-resistant isolates, segregants lacking *sacB* plasmid pAKE604 were identified and verified by plating on LB agar containing 5% sucrose. Isolates were demonstrated to contain the expected mutations by PCR amplification and nucleotide sequencing of *rpoB*.

### GE-resistant mutants: determination of resistance levels

Resistance levels of GE-resistant mutants were quantified by performing broth microdilution assays. Single colonies were inoculated into 5 ml LB broth containing 200 μg/ml ampicillin, and 1 mM IPTG (for *E. coli* plasmid-borne mutants and controls), 5 ml LB broth (for *E. coli* chromosomal mutants and controls), or 5 ml TH broth (for *S. pyogenes* mutants and controls) and incubated at 37°C with shaking in air (for *E. coli*) or in 7% CO_2_/6% O_2_/4% H_2_/83% N_2_ (for *S. pyogenes*) until OD_600_ = 0.4–0.8. Diluted aliquots (∼4 × 10^5^ cells in 50 μl of the same medium) were dispensed into wells of a 96-well plate containing 50 μl of the same medium or 50 μl of a twofold dilution series of GE in the same medium (final concentrations = 0 and 8–8000 μg/ml), and were incubated 16 hr at 37°C with shaking under the same conditions. The MIC was defined as the lowest tested concentration of GE that inhibited bacterial growth by ≥90%.

### GE-resistant mutants: determination of cross-resistance levels

Cross-resistance levels were determined analogously to resistance levels. Liquid cultures were prepared as described above for determination of resistance levels. Diluted aliquots of cultures (∼2 × 10^5^ cells in 97 μl growth medium) were dispensed into wells of a 96-well plate, were supplemented with 3 μl methanol or 3 μl of a twofold dilution series of Rif, Sor, Stl, CBR703, Myx, or Lpm in methanol (final concentrations = 0 and 0.012–50 μg/ml), and were incubated 16 hr at 37°C with shaking.

### Formation of RNAP-promoter open complex

Reaction mixtures contained (20 μl): test compound (0 or 0.5 μM GE, or 2.2 μM Rif), 40 nM *E. coli* RNAP holoenzyme, 10 nM DNA fragment containing positions −42 to +426 of the *lacUV5(ICAP)* promoter (Naryshkin et al., 2001), and 100 μg/ml heparin, in TB. Reaction components other than DNA and heparin were pre-incubated 10 min at 37°C; DNA was added and reaction mixtures were incubated 15 min at 37°C; heparin was added and reactions were incubated 2 min at 37°C to disrupt non-specific RNAP-promoter complexes and RNAP-promoter closed complexes (Cech and McClure, 1980). Products were applied to 5% TBE polyacrylamide slab gels (Bio-Rad, Hercules, CA), gels were electrophoresed in TBE (90 mM Tris-borate, pH 8.0, and 2 mM EDTA), and gels were stained with SYBR Gold Nucleic Acid Gel Stain (Life Technologies).

### Nucleotide addition in transcription initiation: primer-dependent initiation

Reaction mixtures contained (20 μl): test compound (0 or 0.5 μM GE, or 2.2 μM Rif), 5 nM *E. coli* RNAP holoenzyme [Epicentre], 2.5 nM DNA fragment containing positions −49 to +30 of the *lacCONS* promoter (Mukhopadhyay et al., 2001), 25 μg/ml heparin, 500 μM ApA, and 25 μM [α^32^P]UTP (0.9 Bq/fmol) in TB. Reaction components other than DNA, heparin, ApA, and [α-^32^P]UTP were pre-incubated 10 min at 37°C; DNA was added and reaction mixtures were incubated 15 min at 37°C; heparin was added and reaction mixtures were incubated 2 min at 37°C; ApA and [α^32^P]UTP were added and reaction mixtures were incubated 10 min at 37°C. Reactions were terminated by adding 10 μl 80% formamide, 10 mM EDTA, 0.04% bromophenol blue, 0.04% xylene cyanol, and 0.08% amaranth red. Products were heated 5 min at 90°C, cooled 5 min on ice, applied to 16% polyacrylamide (19:1 acrylamide:bisacrylamide, 7 M urea) slab gels, electrophoresed in TBE, and analyzed by storage-phosphor scanning (Typhoon; GE Healthcare, Piscataway, NJ). Identities of tri- and tetranucleotide abortive products from transcription initiation at lacUV5 were defined as in Borowiec and Gralla (1985).

### Nucleotide addition in transcription initiation: de novo initiation

Reaction mixtures contained (20 μl): 0 or 0.5 μM GE, 100 nM *E. coli* RNAP holoenzyme, 20 nM DNA fragment containing positions −65 to +35 of the bacteriophage T7 A1 promoter (prepared by PCR amplification of a synthetic nontemplate-strand oligodeoxyribonucleotide), 25 μg/ml heparin, 25 μM ATP, and 25 μM [α^32^P]UTP (0.7 Bq/fmol) in TB. Reaction components other than DNA, heparin, and NTPs were pre-incubated 5 min at 23°C, DNA was added and reaction mixtures were incubated 15 min at 37°C, heparin and NTPs were added were added and incubated 5 min at 37°C. Reactions were terminated by adding 10 μl 80% formamide, 10 mM EDTA, 0.04% bromophenol blue, and 0.04% xylene cyanol. Products were heated 5 min at 95°C, cooled 5 min on ice, and applied to 16% polyacrylamide (19:1 acrylamide:bisacrylamide, 7 M urea) slab gels, electrophoresed in TBE, and analyzed by storage-phosphor scanning (Typhoon; GE Healthcare).

### Nucleotide addition in transcription elongation: halted elongation complexes

Halted transcription elongation complexes (halted at position +29) were prepared essentially as in Revyakin et al. (2006). Reaction mixtures (18 μl) contained: 40 nM *E. coli* RNAP holoenzyme, 10 nM DNA fragment N25-100-tR2 (Revyakin et al., 2006), 100 μg/ml heparin, 5 μM ATP, 5 μM GTP, and 5 μM [α^32^P]UTP (4 Bq/fmol) in TB. Reaction components except heparin and NTPs were pre-incubated 10 min at 37°C; heparin was added and reaction mixtures were incubated 2 min at 37°C; NTPs were added and reaction mixtures were incubated 5 min at 37°C. The resulting halted transcription elongation complexes were exposed to test compounds by addition of 1 μl 10 μM GE or 1 μl 44 μM Rif, incubated 5 min at 37°C, and were re-started by addition of 1 μl 1 mM CTP and incubation 5 min at 37°C. Reactions were terminated by adding 10 μl 80% formamide, 10 mM EDTA, 0.04% bromophenol blue, 0.04% xylene cyanol, and 0.08% amaranth red. Products were heated 5 min at 90°C, cooled 5 min on ice, applied to 16% polyacrylamide (19:1 acrylamide:bisacrylamide, 7 M urea) slab gels, electrophoresed in TBE, and analyzed by storage-phosphor scanning (Typhoon; GE Healthcare).

### Nucleotide addition in transcription elongation: reconstituted elongation complexes

Nucleic-acid scaffolds for assays were prepared as follows: nontemplate-strand oligodeoxyribonucleotide (5′-TCGCCAGACAGGG-3′; 1 μM), template-strand oligodeoxyribonucleotide (5′-CCCTGTCTGGCGATGGCGCGCCG-3′; 1 μM), and ^32^P-5′-end-labelled oligoribonuceotide (5′-^32^P-CGGCGCGCC-3′; 1 μM; 200 Bq/fmol) in 25 μl 5 mM Tris–HCl, pH 7.7, 200 mM NaCl, and 10 mM MgCl_2_, were heated 5 min at 95°C and cooled to 4°C in 2°C steps with 1 min per step using a thermal cycler (Applied Biosystems) and then were stored at −20°C.

Reaction mixtures for assays contained (15 μl): 0 or 0.5 μM GE or 0 or 2.2 μM Rif, 40 nM wild-type *E. coli* RNAP core enzyme (Epicentre, Madison, WI), 10 nM ^32^P-labelled nucleic-acid scaffold (200 Bq/fmol), and 20 μM ATP in TB. Reaction components except inhibitors and ATP were pre-incubated 5 min at 37°C, GE or Rif was added and reaction mixtures were incubated 5 min at 37°C, and ATP was added and reaction mixtures were incubated 2 min at 37°C. Reactions were terminated by adding 15 μl 80% formamide, 10 mM EDTA, 0.04% bromophenol blue, and 0.04% xylene cyanol, and heating 2 min at 95°C. Products were applied to 20% polyacrylamide (19:1 acrylamide:bisacrylamide, 7 M urea) slab gels, electrophoresed in TBE, and analyzed by storage-phosphor scanning (Typhoon; GE Healthcare).

### RNAP-Rif interaction assays

RNAP-Rif interaction assays were performed as in Feklistov et al. (2008). The assays monitored the quenching of the fluorescence emission of fluorescein incorporated into RNAP holoenzyme at σ^70^ residue 517 (serving as a fluorescence-resonance-energy-transfer donor) by the naphthyl moiety of Rif (serving as a fluorescence-resonance-energy-transfer acceptor; Knight et al., 2005; Feklistov et al., 2008). Fluorescence measurements were performed using a QuantaMaster QM1 spectrofluorometer (PTI, Edison, NJ) (excitation wavelength = 480 nm; emission wavelength = 530 nm; and excitation and emission slit widths = 5 nm).

For determination of association kinetics, 720 μl 2 nM [F^517^]σ^70^-RNAP holoenzyme and 0-2 μM GE in 40 mM Tris–HCl, pH 8.0, 100 mM NaCl, 10 mM MgCl_2_, 1 mM DTT, 0.02% Tween-20, and 5% glycerol was incubated 15 min at 24°C and then mixed with 30 μl 0.01–0.5 μM Rif in the same buffer at 24°C in a cuvette chamber with a mixing dead time ∼0.5 s, and fluorescence emission intensities were monitored for 30 min at 24°C. On-rates for RNAP-Rif interaction, k_on_, were calculated by fitting data to:

$${\text{I}=(\text{I}}_{0}{-\text{I}}_{\infty }{)\text{exp}(-\text{k}}_{\text{obs}}{\text{t})+\text{I}}_{\infty }$$

where k_obs_ is the observed association rate constant at a specified Rif concentration, I is the fluorescence emission intensity at time t, I_o_ is the fluorescence emission intensity at t = 0, and I_∞_ is the fluorescence emission intensity at t = ∞; followed by fitting the Rif-concentration-dependence of k_obs_ to:

$${\text{k}}_{\text{obs}}{=\text{k}}_{\text{on}}{\left[\text{Rif}\right]+\text{k}}_{\text{off}}$$

where k_off_ is ≥0 but is otherwise unconstrained.

For determination of dissociation kinetics, 720 μl of 2 nM [F^517^]σ^70^-RNAP holoenzyme and 0.05 μM Rif in the same buffer was incubated 30 min at 24°C and then mixed with 30 μl of 0–50 μM GE and 12.5–50 μM Sor (which binds to the same site as Rif but does not quench fluorescence emission and therefore serves as a ‘competitor trap’ for Rif dissociation kinetics; Feklistov et al., 2008) in the same buffer at 24°C in a cuvette chamber with a mixing dead time ∼0.5 s; and fluorescence emission intensities were monitored for 5–300 min at 24°C. Dissociation kinetics were found not to depend on the concentration of Sor in the concentration range used in this work (final concentrations of 0.5–2 μM), verifying that Sor in this concentration range does not compete with GE and does not actively displace Rif from RNAP. Off-rates for RNAP-Rif interaction, k_off_, were calculated as:

$${\text{I}=\text{I}}_{0}{+(\text{I}}_{\infty }{-\text{I}}_{\text{0}}{)[\text{1}-\text{exp}(-\text{k}}_{\text{off}}\text{t})]$$

where I is the fluorescence emission intensity at time t, I_o_ is the fluorescence intensity at t = 0, and I_∞_ is the fluorescence intensity at t = ∞.

Equilibrium dissociation constants for RNAP-Rif interaction, K_d_, were calculated as k_off_/k_on_.

The equilibrium dissociation constant for RNAP-GE interaction, K_i_, was calculated from the association-kinetics data, by fitting the GE-concentration-dependence of I_∞_ to:

$${\text{I}}_{\infty }={(\text{I}}_{\infty \text{,max}}[\text{GE}])/{(\text{K}}_{\text{i}}+[\text{GE}])$$

### Structure determination: RNAP + GE

Crystallization and crystal handling were performed essentially as in Tuske et al. (2005). A crystallization stock solution was prepared by adding 1 μl *T. thermophilus* RNAP holoenzyme (10 mg/ml) in 20 mM Tris–HCl, pH 7.7, 100 mM NaCl, and 1% glycerol to 1 μl 33 mM magnesium formate containing 40 μM ZnCl_2_. The crystallization stock solution was equilibrated against a reservoir solution of 30 mM sodium citrate, pH 5.4, and 35 mM magnesium formate in a vapor-diffusion hanging-drop crystallization tray (Hampton Research, Aliso Viejo, CA) at 22°C. Hexagonal crystals formed and grew to a final size of ∼0.4 × ∼0.4 × ∼0.2 mm within 6 d.

GE was soaked into RNAP crystals by addition of 0.2 μl 10 mM GE in 60% (vol/vol) (±)-2-methyl-2,4-pentanediol (MPD; Hampton Research) to the crystallization drop and incubation 15 min at 22°C. Crystals were transferred to solutions containing 0.5 mM GE, 20 mM MES, pH 6.0, 13 mM magnesium formate, 2 mM spermine, 2 mM DTT, 5% PEG400, and 15% (vol/vol) (2R,3R)-(−)-2,3-butanediol (Sigma–Aldrich), and were flash-cooled with liquid nitrogen.

Diffraction data for RNAP-GE were collected at Cornell High Energy Synchrotron Source (CHESS) beamline F1 and were processed and scaled using iMOSFLM and SCALA (Battye et al., 2011; Evans, 2006). The structure of RNAP-GE was solved by molecular replacement with AutoMR in Phenix (McCoy et al., 2007) using a modified structure of *T. thermophilus* RNAP holoenzyme (PDB 3DXJ; Mukhopadhyay et al., 2008) as the search model. Early stages of refinement of the RNAP-GE complex included rigid-body refinement of subdomains (∼15–200 residue segments) of the RNAP molecule. Cycles of rigid-body, individual-atom, and individual-B-factor refinement using Ramachandran and secondary structure restraints and optimized weights for stereochemistry and optimized atomic displacement parameters were carried out using Phenix (Adams et al., 2010). Manual rebuilds against electron-density maps were performed using Coot (Emsley et al., 2010) and Molprobity (Davis et al., 2007; Chen et al., 2010). In addition, two refinement cycles were performed within Autobuster (Bricogne et al., 2011). For GE backbone atoms and GE sidechain atoms with previously defined stereochemistry (Marazzi et al., 2005), an initial atomic model was generated using Maestro (Schrodinger, Portland, OR) and was fit to mFo-DFc maps using Phenix (Adams et al., 2010). For GE sidechain atoms with previously undefined stereochemistry, stereochemistry was deduced and atoms were added based on assessment of mFo-DFc maps and RNAP-GE interactions using PrimeX (Schrodinger). All GE atoms could be fitted to density except atoms of the GE dmaDap residue distal to the sidechain carbonyl moiety. Subsequent cycles of refinement and model building were performed, leading to the current crystallographic model, with a standard crystallographic residual of R_work_ = 0.21 and R_free_ = 0.24 computed using all data from 38.97 to 3.35 Å resolution. Atomic coordinates and structure factors for RNAP-GE have been deposited in the PDB with accession code 4MQ9.

### Structure determination: RP_o_ + GE

Crystals of *T. thermophilus* RP_o_ were prepared using the same nucleic-acid scaffold as used for analysis of RP_o_ in Zhang et al. (2012), and were grown and handled essentially as in Zhang et al. (2012). Crystallization drops contained 1 μl RP_o_ in 20 mM Tris–HCl, pH 7.7, 100 mM NaCl, and 1% glycerol, and 1 μl reservoir buffer (RB; 100 mM Tris–HCl, pH 8.4, 200 mM KCl, 50 mM MgCl_2_, and 9.5% PEG4000), and were equilibrated against 400 μl RB in a vapor-diffusion hanging-drop tray. Rod-like crystals appeared in 1 d, and were used to micro-seed hanging drops using the same conditions.

GE was soaked into RP_o_ crystals by addition of 0.2 μl 20 mM GE in RB to the crystallization drop and incubation 15 min at 22°C. Crystals were transferred in stepwise fashion to successive reservoir solutions containing 1 mM GE in 0.5%, 1%, 2.5%, 5%, 10%, 14%, and 17.5% (v/v) (2R, 3R)-(−)-2,3-butanediol (20 s for first step and 2 s for each subsequent step) and were flash-cooled with liquid nitrogen.

Diffraction data were collected at CHESS beamline F1 and Brookhaven National Laboratory (BNL) beamline X29A and were processed using HKL2000 (Otwinowski and Minor, 1997). Structure factors were converted using the French-Wilson algorithm in Phenix (French and Wilson, 1978) and were subjected to anisotropy correction using the UCLA MBI Diffraction Anisotropy server (Strong et al., 2006; http://services.mbi.ucla.edu/anisoscale/). The structure was solved by molecular replacement with Molrep (Vagin and Teplyakov, 1997) using one RNAP molecule from the structure of *T. thermophilus* RP_o_ (PDB 4 G7H; Zhang et al., 2012) as the search model. Early-stage refinement included rigid-body refinement of the RNAP molecule, followed by rigid-body refinement of each subunit of RNAP molecule. Cycles of iterative model building with Coot (Emsley et al., 2010) and refinement with Phenix (Adams et al., 2010) were performed. Atomic models of the DNA nontemplate strand, the DNA template strand, and GE were built into mFo-DFc omit maps, and subsequent cycles of refinement and model building were performed. The final crystallographic model of RP_o_-GE, refined to R_work_ and R_free_ of 0.21 and 0.25, has been deposited in the PDB with accession code 4OIN.

### Structure determination: RP_o_ + ATP + CMPcPP

ATP (Sigma–Aldrich) and CMPcPP (Jena Biosciences, Jena, Germany) were soaked into RP_o_ crystals (prepared as described above, using the nucleic-acid scaffold used for analysis of RP_o_-GpA in Zhang et al. 2012) by addition of 0.2 μl 30 mM ATP and 30 mM CMPcPP in 55% (vol/vol) RB to the crystallization drop, and incubation 15–20 min at 22°C. Crystals were transferred into reservoir solutions containing 2 mM ATP and 2 mM CMPcPP in 17.5% (vol/vol) (2R, 3R)-(−)-2,3-butanediol and were flash-cooled with liquid nitrogen.

Diffraction data were collected at BNL beamline X25, processed and scaled using HKL2000 (Otwinowski and Minor, 1997), and subjected to anisotropic correction using the UCLA MBI Diffraction Anisotropy server (Strong et al., 2006; http://services.mbi.ucla.edu/anisoscale/). The structure was solved and refined using procedures analogous to those described above for RP_o_-GE. The final crystallographic model contained RP_o_, ATP bound in the RNAP i site, and CMPcPP:Mg^2+^ bound in the RNAP i+1 site. The final crystallographic model of RP_o_-ATP-CMPcPP, refined to R_work_ and R_free_ of 0.21 and 0.26, respectively, has been deposited in the PDB with accession code 4OIO.

### Structure determination: RP_o_ + GE + ATP + CMPcPP

Crystals of RP_o_ (prepared as described above for RP_o_ + ATP + CMPcPP) first were soaked with GE (addition of 0.2 μl 20 mM GE in RB to the crystallization drop and incubation 15 min at 22°C) and then were soaked with ATP and CMPcPP (addition of 0.2 μl 30 mM ATP and 30 mM CMPcPP in 55% [vol/vol] RB to the crystallization drop and incubation 15 min at 22°C). Crystals then were transferred to reservoir solutions containing 1 mM GE, 2 mM ATP, and 2 mM CMPcPP in 17.5% (vol/vol) (2R, 3R)-(−)-2,3-butanediol and were flash-cooled with liquid nitrogen.

Diffraction data were collected at BNL beamline X25, and were processed, scaled, and corrected for anisotropy using HKL2000 (Otwinowski and Minor, 1997). The structure was solved and refined using procedures analogous to those described above for RP_o_-GE. The final crystallographic model contained RP_o,_ GE bound to the GE target, and ATP:Mg^2+^ bound to the RNAP E site, and did not contain ATP in the RNAP i site or CMPcPP in RNAP i+1 site. The final crystallographic model, refined to R_work_ and R_free_ of 0.21 0.25, respectively, has been deposited in the PDB with accession code 4OIP.

### Structure determination: RP_o_ + GE + Rif

Crystals of RP_o_ (prepared as described above for RP_o_ + ATP + CMPcPP) first were soaked with Rif (addition of 0.1 μl 20 mM Rif in RB containing 40% [vol/vol] [2R, 3R]-(−)-2,3-butanediol to the crystallization drop and incubation 15 min at 22°C) and then were soaked with GE (addition of 0.2 μl 20 mM GE in RB to the crystallization drop and incubation 15 min at 22°C). Crystals then were transferred to reservoir solutions containing 1 mM GE and 0.4 mM Rif in 17.5% (vol/vol) (2R, 3R)-(−)-2,3-butanediol and were flash-cooled with liquid nitrogen.

Diffraction data were collected at CHESS beamline F1, and were processed and scaled using HKL2000 (Otwinowski and Minor, 1997). The structure was solved and refined using procedures analogous to those described above for RP_o_-GE. The final crystallographic model contained RP_o_ and GE bound to the GE target but did not contain Rif. The final crystallographic model, refined to R_work_ and R_free_ of 0.20 and 0.25, respectively, has been deposited in the PDB with accession code 4OIQ.

### Structure determination: RP_o_ + GE + RifSV

Crystals of RPo (prepared as described above for RP_o_ + ATP + CMPcPP) first were soaked with RifSV (addition of 0.2 μl 10 mM RifSV in RB to the crystallization drop and incubation 15 min at 22°C, or transfer of the crystal to 1 μl 10 mM RifSV in RB and incubation 15 min at 22°C) and then were soaked with GE (addition of 0.2 μl 20 mM GE in RB to the drop and incubation 15 min at 22°C). Crystals then were transferred in to reservoir solutions containing 1 mM GE and 1 mM RifSV in 17.5% (vol/vol) (2R, 3R)-(−)-2,3-butanediol and were flash-cooled with liquid nitrogen.

Diffraction data were collected at BNL beamline X25, were processed and scaled using HKL2000 (Otwinowski and Minor, 1997), and were subjected to anisotropic correction using the UCLA MBI Diffraction Anisotropy server (Strong et al., 2006; http://services.mbi.ucla.edu/anisoscale/). The structure was solved and refined using procedures analogous to those described above for RP_o_-GE. The final crystallographic model contained RP_o_, GE bound to the GE target, and RifSV bound to the Rif target. The final crystallographic model of RP_o_-GE-RifSV, refined to R_work_ and R_free_ of 0.21 and 0.25, respectively, has been deposited in the PDB with accession code 4OIR.

### Synthesis of a GE-rifamycin bipartite inhibitor: step 1, synthesis of [ζ^1^-amino-dmaDap; α-descarboxy-Ama]GE (compound 1 of Figure 7C)

**Figure:**
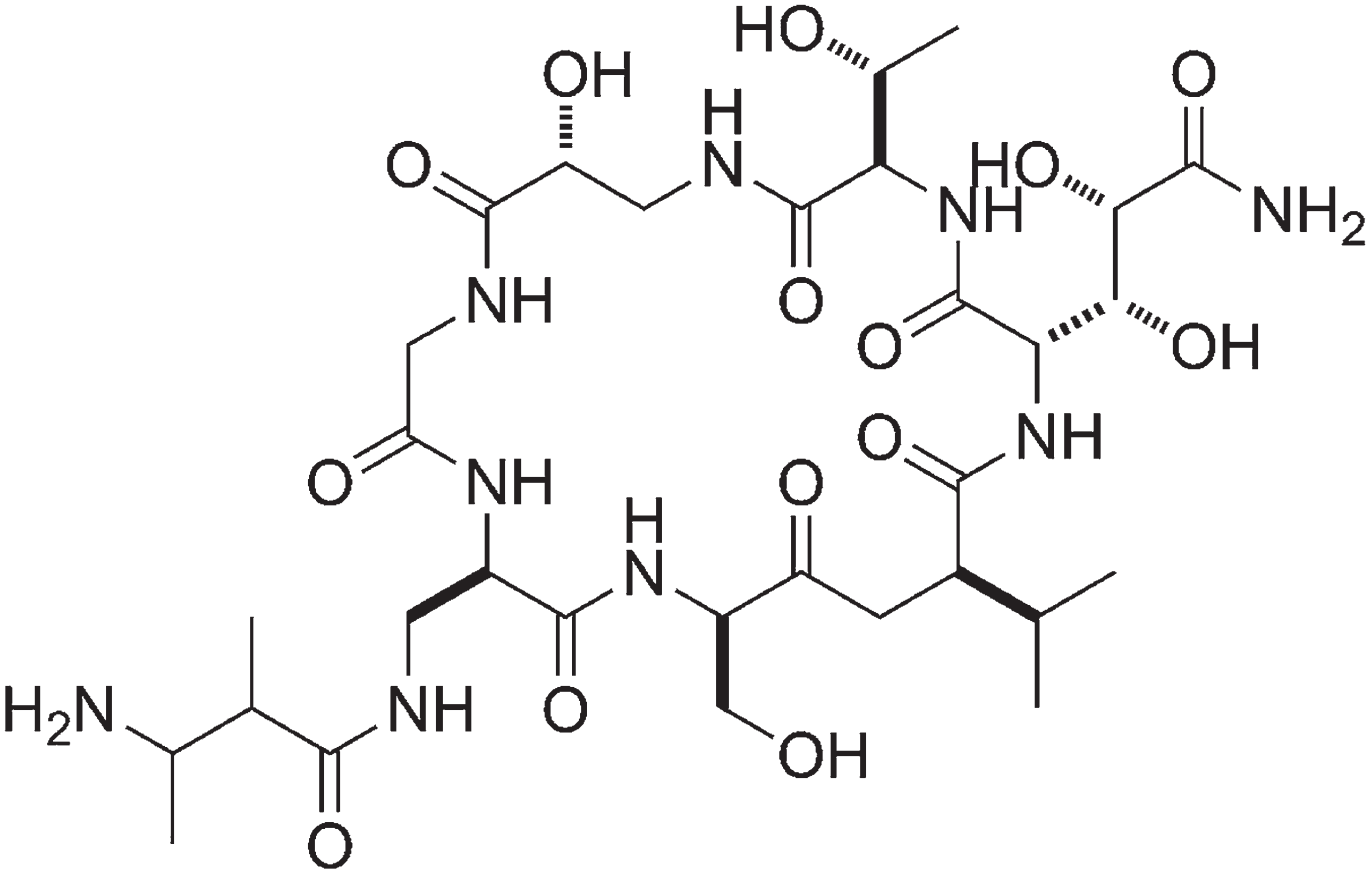

GE (20 mg 25 μmol), ammonium acetate (60 mg; 780 μmol; Aldrich), and perchloric-acid-impregnated silica (5 mg; prepared as in Singh et al., 2009), were mixed in 4 ml absolute ethanol in a screw-cap vial. The mixture was microwaved for 4 × 30 s (1000 W) with intervals of 1 min for re-mixing contents of the vial. The mixture was allowed to incubate at room temperature for another 16 hr, evaporated to dryness, and resuspended in 2 ml 1% triethylamine-water. The mixture was centrifuged, and the supernatant was purified via HPLC (Phenomenex C18, semi-prep; 5 min 0% B, 20 min 5% B, 25 min 10% B, 30 min 30% B, 40 min 80% B; A = water, B = acetonitrile, 2 ml/min).

The HPLC elution profile and mass spectrum of the product indicate that the product has undergone decarboxylation of the Ama sidechain (Mariani et al., 2005). It is known that acid and heat induce decarboxylation of the GE Ama sidechain, and that decarboxylated GE exhibits ∼1/20 the RNAP-inhibitory activity and antibacterial activity of GE (Mariani et al., 2005).

Yield: 3.5 mg; 18%.

MS (MALDI): calculated: *m/z* 777.80 (MH^+^); found: 778.20, 800.59 (M + Na^+^).

### Synthesis of GE-rifamycin bipartite inhibitor: step 2, synthesis of {[α-descarboxy-Ama]GE}-NH-{rifamycin S} (compound 2 of Figure 7C)

**Figure:**
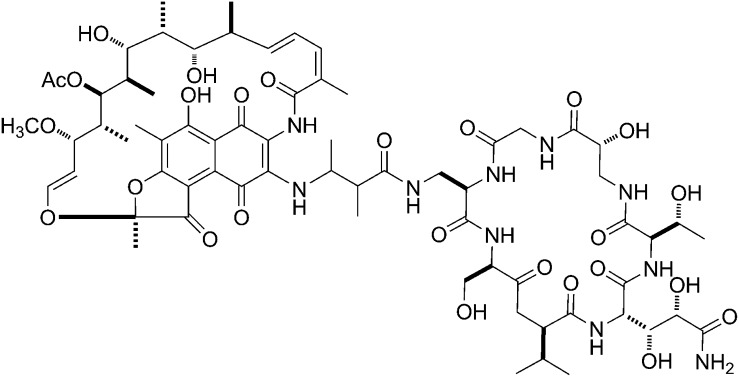

3-Bromo-rifamycin S (2.7 mg; 3.47 μmol; prepared as in Marchi and Montecchi 1979), compound 1 (2.7 mg; 3.47 μmol; Example 1a) and triethylamine (0.5 μl; 3.47 μmol; Aldrich) were mixed together in 200 μl DMF and allowed to react for 18 hr at 25°C. The reaction mixture was quenched with 100 μl water, centrifuged, and the supernatant was purified via HPLC (Phenomenex C18, semi-prep; 0 min 10% B, 35 min 100% B; A = water, B = acetonitrile, 2 ml/min).

Yield: 1.51 mg; 30%.

MS (MALDI): calculated: *m/z* 1493.52 (M + Na^+^); found: 1494.22.

### Synthesis of GE-rifamycin bipartite inhibitor: step 3, synthesis of {[α-descarboxy-Ama]GE}-NH-{RifSV} (compound 3 of Figure 7C; “RifaGE-3”)

**Figure:**
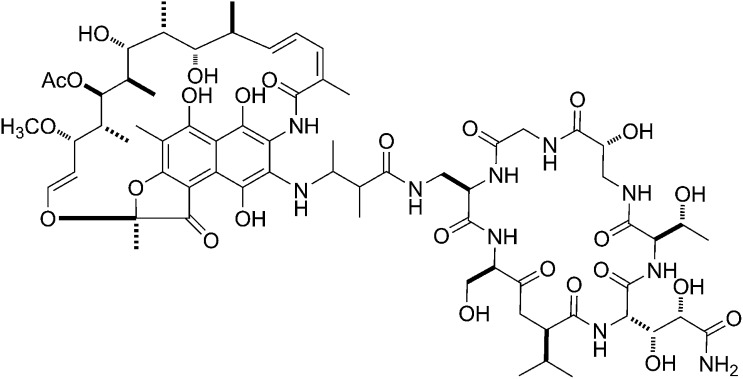

Sodium ascorbate (2.38 mg; 12 μmol; Aldrich) in 25 μl water was added to compound 2 (0.600 mg; 0.4 μmol; Example 1b) in 100 μl water, mixed, and allowed to react for 10 min at 25°C. The product was isolated via HPLC (Phenomenex C18, analytical; 0’ 10% B, 35′ 100% B; A = water, B = acetonitrile, 1 ml/min).

Yield: 0.1 mg; 17%.

MS (MALDI): calculated: m/z 1495.52 (M + Na+); found: 1495.71.

## Additional files

Supplementary file 1. (**A**) GE: antibacterial activity. (**B**) GE: RNAP-inhibitory activity. **DOI:** http://dx.doi.org/10.7554/eLife.02450.021

Supplementary file 2. (**A**) ‘Doped’ oligonucleotide primers used for saturation mutagenesis. (**B**) Chromosomal GE^R^ mutants in *E. coli* D21f2tolC: sequences and properties. (**C**) Chromosomal GE^R^ mutants in *S. pyogenes*: sequences and properties. (**D**) Chromosomal GE^R^ mutants in *E. coli* D21f2tolC: absence of cross-resistance to Rif. (**E**) Chromosomal Rif^R^ mutants in *E. coli* D21f2tolC: absence of cross-resistance to GE. (**F**) GE^R^ mutants from saturation mutagenesis: absence of cross-resistance to Sor, Stl, CBR703, Myx, and Lpm. **DOI:** http://dx.doi.org/10.7554/eLife.02450.022

### Major datasets

The following datasets were generated:

HoMX, ArnoldE, EbrightRH, ZhangY, and TuskeS, 2013, Crystal structure of *Thermus thermophilus* RNA polymerase holoenzyme in complex with GE23077, http://www.rcsb.org/pdb/explore/explore.do?structureId=4MQ9, Publicly available at RCSB Protein Data Bank.

ZhangY, ArnoldE, and EbrightRH, 2014, Crystal structure of *Thermus thermophilus* transcription initiation complex soaked with GE23077, http://www.rcsb.org/pdb/explore/explore.do?structureId=4OIN, Publicly available at RCSB Protein Data Bank.

ZhangY, ArnoldE, and EbrightRH, 2014, Crystal structure of *Thermus thermophilus* pre-insertion substrate complex for de novo transcription initiation, http://www.rcsb.org/pdb/explore/explore.do?structureId=4OIO, Publicly available at RCSB Protein Data Bank.

ZhangY, ArnoldE, and EbrightRH, 2014, Crystal structure of *Thermus thermophilus* transcription initiation complex soaked with GE23077, ATP, and CMPcPP, http://www.rcsb.org/pdb/explore/explore.do?structureId=4OIP, Publicly available at RCSB Protein Data Bank.

ZhangY, ArnoldE, and EbrightRH, 2014, Crystal structure of *Thermus thermophilus* transcription initiation complex soaked with GE23077 and rifampicin, http://www.rcsb.org/pdb/explore/explore.do?structureId=4OIQ, Publicly available at RCSB Protein Data Bank.

ZhangY, ArnoldE, and EbrightRH, 2014, Crystal structure of *Thermus thermophilus* RNA polymerase transcription initiation complex soaked with GE23077 and rifamycin SV, http://www.rcsb.org/pdb/explore/explore.do?structureId=4OIR, Publicly available at RCSB Protein Data Bank.

**Reporting standards:** Standard used to collect data: http://www.wwpdb.org/policy.html.
