# Supplementary material for: GE23077 binds to the RNA polymerase ‘i’ and ‘i+1’ sites and prevents the binding of initiating nucleotides
Source: eLife. 2014 Apr 22;3:e02450. doi: 10.7554/eLife.02450 (PMC3994528; doi:10.7554/eLife.02450)
Supplement: Supplementary file 1. — (A) GE: antibacterial activity. (B) GE: RNAP-inhibitory activity. DOI: http://dx.doi.org/10.7554/eLife.02450.021 [file elife02450s001.doc]

**Supplementary file 1A.**

**GE: antibacterial activity**

| **organism** | **MIC (µg/ml)** |
| --- | --- |
|  |  |
| **Gram-negative bacteria** |  |
| *Moraxella catarrhalis* ATCC 25238 | 25 |
| *Escherichia coli* D21f2tolC | 250 |
|  |  |
| **Gram-positive bacteria** |  |
| *Streptococcus pyogenes* ATCC 12344 | 250 |
|  |  |

**Supplementary file 1B.**

**GE: RNAP-inhibitory activity**

| **RNAP** | **IC50 (µM)** |
| --- | --- |
|  |  |
| **Gram-negative bacterial RNAP** |  |
| *Escherichia coli* RNAP | 0.02 |
|  |  |
| **Gram-positive bacterial RNAP** |  |
| *Staphylococcus aureus* RNAP | 0.02 |
|  |  |
| **Mycobacterial RNAP** |  |
| *Mycobacterium tuberculosis* RNAP | 0.1 |
|  |  |
| **Thermus/Deinococcus clade RNAP** |  |
| *Thermus thermophilus* RNAP | 20 |
|  |  |
| **Human RNAP I / II / III** |  |
| HeLa nuclear extract | >50 |
|  |  |
