# Supplementary material for: GE23077 binds to the RNA polymerase ‘i’ and ‘i+1’ sites and prevents the binding of initiating nucleotides
Source: eLife. 2014 Apr 22;3:e02450. doi: 10.7554/eLife.02450 (PMC3994528; doi:10.7554/eLife.02450)
Supplement: Supplementary file 2. — (A) ‘Doped’ oligonucleotide primers used for saturation mutagenesis. (B) Chromosomal GER mutants in E. coli D21f2tolC: sequences and properties. (C) Chromosomal GER mutants in S. pyogenes: sequences and properties. (D) Chromosomal GER mutants in E. coli D21f2tolC: absence of cross-resistance to Rif. (E) Chromosomal RifR mutants in E. coli D21f2tolC: absence of cross-resistance to GE. (F) GER mutants from saturation mutagenesis: absence of cross-resistance to Sor, Stl, CBR703, Myx, and Lpm. DOI: http://dx.doi.org/10.7554/eLife.02450.022 [file elife02450s002.doc]

**Supplementary file 2A. “Doped” oligonucleotide primers used for saturation mutagenesis**

| codons | sequence |
| --- | --- |
|  |  |
| ***rpoC*** |  |
|  |  |
| 347-355^b^ | 5’-cggtaagcgtGTTGACTACTCCGGTCGTTCTGTAATCaccgtaggtcC-3’ |
|  |  |
| 425-429^a^ | 5’-gtactgctgaacCGTGCACCGACTCTGcaccgtctggG-3’ |
|  |  |
| 456-465^b^ | 5’-gtttgtgcgGCATATAACGCCGACTTCGATGGTGACCAGatggctgttc-3’ |
|  |  |
| 779-792^b^ | 5’‑ccacccacggtGCTCGTAAAGGTCTGGCGGATACCGCACTGAAAACTGCGAACtccggtta cc-3’ |
|  |  |
| 934-943^b^ | 5’-gctgaccatgcgtACGTTCCACATCGGTGGTGCGGCATCTCGTgcggctgctg-3’ |
|  |  |
| ***rpoB*** |  |
|  |  |
| 136-143^b^ | 5’-cagacaacggtaccTTTGTTATCAACGGTACTGAGCGTgttatcgtttccc-3’ |
|  |  |
| 504-511^b^ | 5’-ccgcagcagtgaaaGAGTTCTTCGGTTCCAGCCAGCTGtctcagtttatggacc-3’ |
|  |  |
| 512-522^b^ | 5’-CcagccagctgTCTCAGTTTATGGACCAGAACAACCCGCTGTCTgagattacgCAC-3’ |
|  |  |
| 523-534^b^ | 5’-cccgctgtctGAGATTACGCACAAACGTCGTATCTCCGCACTCGGCccaggcggtc-3’ |
|  |  |
| 535-541^b^ | 5’-ccgcactcggcCCAGGCGGTCTGACCCGTGAAcgtgcaggcttc-3’ |
|  |  |
| 542-549^b^ | 5’-ctgacccgtgaaCGTGCAGGCTTCGAAGTTCGAGACgtacacccg-3’ |
|  |  |
| 563-573^b^ | 5’-ccaatcgaaACCCCTGAAGGTCCGAACATCGGTCTGATCAACtctctgtccg-3’ |
|  |  |
| 677-690^b^ | 5’‑gatgacgccAACCGTGCATTGATGGGTGCGAACATGCAACGTCAGGCCGTTccgactctg-3’ |
|  |  |
| 758-763^a^ | 5’-gaccaaatacaccCGTTCTAACCAGAACACCtgtatcaaccag-3’ |
|  |  |
| 813-814^a^ | 5’-ggttacaacttcGAAGACtccatcctcg-3’ |
|  |  |
| 829-835^b^ | 5’-caggaagaccgtttcACCACCATCCACATTCAGGAActggcgtgtgtg-3’ |
|  |  |
| 1054-1060^b^ | 5’-gttaaggtatatCTGGCGGTTAAACGCCGTATCcagcctggtgac-3’ |
|  |  |
| 1064-1074^b^ | 5’-ccagcctggtGACAAGATGGCAGGTCGTCACGGTAACAAGGGTgtaatttctaag-3’ |
|  |  |
| 1102-1108^b^ | 5’-gaacccgctgGGCGTACCGTCTCGTATGAACatcggtcag-3’ |
|  |  |
| 1233-1242^b^ | 5’-catgtacatgCTGAAACTGAACCACCTGGTCGACGACAAGatgcacgcgc-3’ |
|  |  |

^a^ The underlined regions were synthesized using a mixture of 92% of the correct phophoramidite and 8% of a 1:1:1:1 mixture of dA, dC, dG, and dT phosphoramidities at each position.

^b^ The underlined regions were synthesized using a mixture of 98% of the correct phophoramidite and 2% of a 1:1:1:1 mixture of dA, dC, dG, and dT phosphoramidities at each position.

**Supplementary file 2B.**

**Chromosomal GE^R^ mutants in *E. coli* D21f2tolC:**

**sequences and properties**

| **amino acid**  **substitution** | **GE resistance level**  **(MIC/MIC_wild-type_)^a^** |
| --- | --- |
|  |  |
| ***rpoB* (RNAP β subunit)** |  |
|  |  |
| 565 Glu→Asp | >16 |
| 684 Asn→Thr | 16 |
|  |  |

^a^ MIC_wild-type_ = 500 μg/ml.

**Supplementary file 2C.**

**Chromosomal GE^R^ mutants in *S. pyogenes*:**

**sequences and properties**

| **amino acid**  **substitution^a^** | **resistance level**  **(MIC/MIC_wild-type_)^b^** |
| --- | --- |
|  |  |
| ***rpoB* (RNAP β subunit)** |  |
|  |  |
| 565 [525] Glu→Gly | 32 |
| 565 [525] Glu→Val | 32 |
| 681 [643] Met→Lys | 16 |
| 684 [646] Asn→Ile | >32 |
|  |  |

^a^ Residues are numbered as in *E. coli* RNAP and, in brackets, as in *S. pyogenes* RNAP.

^b^ MIC_wild-type_ = 250 μg/ml.

**Supplementary file 2D.**

**Chromosomal GE^R^ mutants in *E. coli* D21f2tolC:**

**absence of cross-resistance to Rif**

| **amino acid**  **substitution** | **MIC ratio**  **(MIC/MIC_wild-type_)^a^** | |
| --- | --- | --- |
|  | **GE** | **Rif** |
|  |  |  |
| ***rpoB* (RNAP β subunit)** |  |  |
|  |  |  |
| 565 Glu→Asp | >16 | 1 |
| 684 Asn→Thr | 16 | 1 |
|  |  |  |

^a^  MIC_wild-type,GE_ = 500 μg/ml; MIC_wild-type,Rif_ = 0.2 μg/ml.

**Supplementary file 2E.**

**Chromosomal Rif^R^ mutants in *E. coli* D21f2tolC:**

**absence of cross-resistance to GE**

| **amino acid**  **substitution** | **MIC ratio**  **(MIC/MIC_wild-type_)^a^** | |
| --- | --- | --- |
|  | **GE** | **Rif** |
|  |  |  |
| ***rpoB* (RNAP β subunit)** |  |  |
|  |  |  |
| 516 Asp→Val | 0.5 | 512 |
| 526 His→Asp | 0.5 | >1024 |
| 526 His→Tyr | 4 | >1024 |
| 531 Ser→Leu | 1 | 1024 |
|  |  |  |

^a^ MIC_wild-type,GE_ = 500 μg/ml; MIC_wild-type,Rif_ = 0.2 μg/ml.

**Supplementary file 2F.**

**GE^R^ mutants from saturation mutagenesis:**

**absence of cross-resistance to Sor, Stl, CBR703, Myx, and Lpm**

| **amino acid**  **substitution** | **MIC ratio (MIC/MIC_wild-type_)^a^** | | | | |
| --- | --- | --- | --- | --- | --- |
|  | **Sor** | **Stl** | **CBR703** | **Myx** | **Lpm** |
|  |  |  |  |  |  |
| ***rpoB* (RNAP β subunit)** |  |  |  |  |  |
|  |  |  |  |  |  |
| 565 Glu→Asp | 1 | 1 | 1 | 1 | 1 |
| 566 Gly→Arg | 1 | 1 | 1 | 1 | 1 |
| 566 Gly→Cys | 1 | 1 | 1 | 1 | 1 |
| 566 Gly→Ser | 1 | 1 | 1 | 1 | 1 |
| 684 Asn→Lys | 1 | 1 | 1 | 1 | 1 |
| 684 Asn→Thr | 1 | 1 | 2 | 1 | 1 |
|  |  |  |  |  |  |

^a^ MIC_wild-type,Sor_ = 1.56 µg/ml; MIC_wild-type,Stl_ = 0.78 µg/ml; MIC_wild-type,CBR703_ = 6.25 µg/ml;

MIC_wild-type,Myx_ = 0.098 µg/ml, MIC_wild-type,Lpm_ = 0.78 µg/ml.
